# Transformation-associated recombination and heterologous expression of noncanonical depsipeptide nonribosomal peptide synthetase derived from marine *Streptomyces*

**DOI:** 10.1007/s42995-025-00296-8

**Published:** 2025-04-24

**Authors:** Jeong Sang Yi, Jin Won Choi, Ngoc Han Le Thi, Sung Jin Kim, Hyun-Ju Kim, Jung Min Kim, Jun Eui Park, Kyuho Moon, Dong Chan Oh, Sang Hee Shim, Ki Sung Kang, Yeo Joon Yoon

**Affiliations:** 1 Natural Products Research Institute, College of Pharmacy, Seoul National University, Seoul, 08826 Republic of Korea; 2 College of Korean Medicine, Gachon University, Seongnam, 13120 Republic of Korea; 3 College of Pharmacy, Kyung Hee University, Seoul, 02447 Republic of Korea

**Keywords:** *Streptomyces*, Depsipeptide, Nonribosomal peptide synthetase, Anti-inflammatory

## Abstract

Analysis of the secondary metabolite biosynthesis gene cluster (BGC) from marine *Streptomyces* sp. SNJ102 revealed the presence of a noncanonical nonribosomal peptide synthetase (NRPS), predicted to produce a depsipeptide compound. The NRPS gene cluster was captured by transformation-associated recombination and heterologously expressed in *Streptomyces albus*. The production of the new compound was confirmed using high-resolution liquid chromatography-mass spectrometry, and its structure was elucidated using nuclear magnetic resonance spectroscopy. The structure of the new depsipeptide was more similar to the monomeric structure of cyclic depsipeptides derived from fungi than to other *Streptomyces*-derived depsipeptides. In addition, the bacterial depsipeptide, which we named jejumide, showed promising anti-inflammatory activity. These results demonstrate that genome mining and successful heterologous expression of cryptic nonlinear NRPS BGCs from marine bacteria will facilitate the discovery of novel nonribosomal peptides and understanding of the complicated biosynthetic mechanism of nonlinear NRPS.

**Supplementary Information:**

The online version contains supplementary material available at 10.1007/s42995-025-00296-8.

## Introduction

*Streptomyces* are responsible for the production of a vast majority of microbial bioactive natural products. Up to 2020, approximately 42% of the bacterial natural products were produced by *Streptomyces* species, with 30% of them belonging to the non-ribosomal peptide (NRP) class (Newman et al. 2020). The importance of secondary metabolites in marine *Streptomyces* is increasing because of their wide distribution over 70% of the Earth’s surface and their ability to survive in diverse environments. In particular, the discovery of numerous NRPs with unprecedented structures has increased the likelihood of discovering new classes of noncanonical nonribosomal peptide synthetase (NRPS) (Fenical and Jensen 2006). NRPs are secondary metabolites synthesized by NRPS and serve as antibiotics, anticancer agents, and toxins (Liu et al. 2019). NRPS modules generally consist of condensation (C), adenylation (A), and peptidyl carrier protein (PCP) domains and are expressed as intact, complete modules. In canonical NRPS assembly lines, the order of the NRPS modules corresponds to the sequence of amino acids in the NRP. The thioesterase (TE) domain of the last module usually releases or cyclizes the product. A growing number of noncanonical or nonlinear NRPS that violate canonical NRP biosynthesis have been reported (Kim et al. 2021, Jaremko et al. 2020, Wenzel et al. 2005, Mootz et al. 2002). To effectively employ such NRPSs for the biosynthesis of bioactive secondary metabolites and understand their biosynthetic mechanisms, both gene clusters and corresponding product structures should be available. However, either one is frequently absent, demanding heterologous expression of nonlinear NRPS genes to access their novel products and identify the biosynthetic pathways. The identification of additional NRP products from nonlinear NRPSs will help in the development of bioinformatic tools for the systematic and efficient prediction of new NRP products from nonlinear NRPSs and understanding their biosynthetic mechanism.

Depsipeptides are NRPs with one or more peptide bonds replaced by ester bonds and are produced by bacteria, fungi, and other organisms. Depsipeptides exhibit various biological activities, including antitumor, anti-inflammatory, and antimicrobial properties (Agata et al. 1995; Takahashi et al. 2001, Wang et al. 2018, Andavan et al. 2010). Fungi produce the largest number of depsipeptides, ranging from tri- to decacyclic depsipeptides (Wang et al. 2018). The majority of fungal depsipeptides have ester bonds formed between the carboxylic acid moiety of amino acids and the α-hydroxy group of α-keto or α-hydroxy acids. As the length of the depsipeptide increases, depsipeptide elongation tends to occur iteratively, providing symmetry to the core structures (Süssmuth et al. 2011). In contrast, ester bond formation in depsipeptides from *Streptomyces* species is more diverse than that in fungi. Ester bond formation occurs only once during the NRP chain-termination step, and is formed between the hydroxy moieties of the serine or threonine side chains and the carboxylic acid moiety of the last amino acid. Therefore, their core structure is highly complex and asymmetric (Takahashi et al. 2001, Makarieva et al. 2022, Hassan et al. 2015).

In this study, we discovered a bacterial depsipeptide compound **1**, which we named jejumide, via heterologous expression of the noncanonical depsipeptide NRPS biosynthetic gene cluster (BGC) identified using genome mining of *Streptomyces* sp. SNJ102 (SNJ102), which was isolated from samples collected from deep-sea sediments (depth = 108 m) off the coast of Sungsanpo, Jeju Island, Republic of Korea. The chemical structure of compound **1** resembled that of the monomeric form of the fungal depsipeptide bassianolide rather than those of previously reported *Streptomyces*-derived depsipeptides (Jirakkakul et al. 2008, Roig et al. 2014). In addition, compound **1** exhibited anti-inflammation activity by inhibiting the expression of cyclooxygenase-2 (COX- 2) and inducible nitric oxide synthase 2 (iNOS). The identification of a novel depsipeptide, BGC, from a marine *Streptomyces* strain highlights the importance of exploring noncanonical NRPS, especially those from marine bacteria; these explorations will facilitate the discovery and production of novel bioactive compounds.

## Materials and methods

### General experimental procedures

The optical rotation was recorded using a JASCO P-2000 polarimeter (JASCO, Easton, PA, USA). High resolution electrospray ionization mass spectrometry (MS) data were acquired with a quadrupole time-of-flight (qToF) mass spectrometer on a qToF 6530 MS/1290 Infinity system (Agilent Technologies, Santa Clara, CA, USA). An Xselect® CSH™ C18 column XP (2.1 mm × 100 mm, 2.5 μm (Waters, Milford, MA, USA)) was used and was maintained at 40 °C. The nuclear magnetic resonance (NMR) spectra were obtained using a Bruker 800 MHz (^1^H: 800 MHz, ^13^C: 200 MHz) spectrometer (Bruker, Billerica, MA, USA) with CD_3_OD (Cambridge Isotope Laboratories, Inc., Tewksbury, MA, USA) and DMSO-*d*_6_ (Cambridge Isotope Laboratories, Inc.).

### Bioinformatic analysis and BAC library construction

antiSMASH5.0 (Blin et al. 2019) was used to analyze the secondary metabolism of *Streptomyces* sp. SNJ102, and BGC maps were drawn using Easyfig version 2.2.5 for Windows (Sullivan et al. 2011). antiSMASH analysis was performed with default settings, and incomplete secondary metabolite BGC and BGC without core PK/NRP modules, where there are only a limited number of individual domains, were excluded from further analysis. Sequence alignments and phylogenetic analysis of C domains were performed using ClustalW (Madeira et al. 2024). The BAC library of *Streptomyces* sp. SNJ102 was constructed by Bio S&T (Montreal, QC, Canada). A total of 4,224 BAC clones were constructed with an average insert size of 100 kb and genome coverage of 52 ×.

### Transformation-associated recombination cloning procedure

The pCB_Apr vector (Kim et al. 2023a) was maintained in *Escherichia coli* DH5α before its usage. It was amplified via polymerase chain reaction (PCR) using primers containing 5′- and 3′-end homologous sequences of the target BGC (Table S1). The BAC library was screened for the SNJ102 depsipeptide BGC via PCR amplification and sequencing of the NRP modules orf- 6211, 6212, 6214, 6215, and 6218 using the primers listed in Table S1. The BAC plasmid containing the target BGC was digested with *Dra*I. Two micrograms of each PCR-amplified pCB_Apr and *Dra*I digested BAC plasmid were transformed into *Saccharomyces cerevisiae* BY4727 using lithium acetate, salmon sperm carrier DNA, and the polyethylene glycol 3350 transformation method described in a previous study (Gietz et al. 2007). *S. cerevisiae* BY4727 carrying BGC captured plasmids were grown on selective agar media without histidine for 4 days at 30 ℃. Yeast colonies were grown in selective liquid media without histidine for 2 days at 30 ℃ with 200 r/min shaking, and the pCB_Apr plasmid containing the target BGC was extracted by the QIAprep® miniprep kit (Qiagen, Hilden, Germany). The resulting pCB_102DP construct was sequenced and verified using the primers listed in Table S1, before transformation into *Streptomyces* heterologous expression host strains. All bacterial strains and vectors used in this study are listed in Table S2.

### Heterologous expression of target BGC and LC–MS analysis

pCB_102DP was maintained in *E. coli* DH10B and *E. coli* ET12567, and conjugated into the *Streptomyces* host strains, *Streptomyces albus* J1074 (Kim et al. 2023a, b), *Streptomyces venezuelae* YJ028 (Han et al. 2011), and *Streptomyces roseosporus* NRRL 11379, by triparental conjugation using *E. coli* DH5α containing pRK2013 (Table S2). *Streptomyces* colonies harboring pCB_102DP were selected on ISP2 media (Becton Dickinson, Franklin Lakes, NJ, USA) containing 100 μg/mL nalidixic acid and 50 μg/mL apramycin for three rounds. R5^−^, ISP2, and TSBY liquid media were used for the initial bacterial cultures, and R5^−^ liquid medium was used primarily for secondary metabolite production (Yi et al. 2020). mRNA was extracted using the Qiagen mRNA isolation kit and reverse transcription PCR was performed using the Qiagen OneStep RT-PCR kit, following the manufacturer’s protocol. *Streptomyces* heterologous hosts were cultured in 50 mL of R5^−^ in a 250 mL baffled flask with 8 g of 3 mm glass beads or in 500 mL of R5^−^ in a 3 L baffled flask with 36 g of 3 mm glass beads at 28 ℃ with 220 r/min shaking for 7 days. Mycelia were harvested via centrifugation at 3200 r/min for 7 min. Equal volumes of ethyl acetate and 80% methanol were used to extract metabolites from culture broth and mycelia, respectively; the extraction was repeated thrice. The extracts were then dried in a rotary vacuum evaporator. Extracts from two sets of 30 L of the bacterial culture were prepared for the isolation, structure elucidation, and bioassays of compound **1**. The weights of the extracts were measured, and 20 μL of 50 μg/mL extracts in acetonitrile was analyzed by qToF LC–MS. A linear gradient from 10 to 100% aqueous CH_3_CN (0.1% formic acid) for 14 min was used as a mobile phase, and electrospray ionization was used as the ionization method.

### Isolation of compound 1

The extract was fractionated via medium-pressure column chromatography over silica using a stepwise gradient of *n*-hexane-methylene chloride-MeOH (100:0:0, 50:50:0, 0:100:0, 0:99:1, 0:98:2, 0:96:4, 0:93:7, 0:90:10, 0:85:15, 0:75:25, 0:50:50, 0:25:75, and 0:0:100) to obtain ten fractions (fractions 1–10). Fraction 9 (20.6 mg) was separated via reversed-phase high-performance liquid chromatography (Phenomenex, Torrance, CA, USA) (Luna C_18_ (2), 5 µ, 250 mm × 10.0 mm, isocratic aqueous 65% ACN, 2.0 mL/min, UV 210, 254, 280, 365 nm) to obtain **1** (0.7 mg).

The characteristics of Jejumide (**1**) are as follows: colorless gel; [α]_D_^25^ = + 46.60 (*c* 0.1 MeOH); UV (MeOH) λ_max_ (log *ε*) 193 (6.69) nm (Table 1); ^1^H–^1^H COSY, H–2 ↔ H–3, H–3 ↔ H–4, H–4 ↔ H_3–_–5, H–4 ↔ H_3_– 6, H–2'↔ H–3', H–3'↔ H_3_– 4', H–3'↔ H_3_– 5', H–2''↔ H–3'', H–3''↔ H_3_– 4'', H–3 ↔ H_3_–5'', H–2'''↔ H–3''', H–3'''↔ H–4''', H–4'''↔ H_3_–5''', H–4 ↔ H_3_–6''''; Total HMBC correlations H–# → C–#, H–2 → C–1, C–3, and C–4, H–3 → C–1, C–2, C–4, C–5, and C–6, H–4 → C–2, C–3, C–5 and C–6, H_3_–5 → C–3, C–4, and C–6, H_3_–6 → C–3, C–4, and C–5, H–2'→ C–1, C–1', C–3', C–4'and C–5', H–3'→ C–1', C–2', C–4', and C–5', H_3_–4'→ C–2', C–3'and C–5', H_3_–5'→ C–2', C–3', and C–4', H–2''→ C–1', C–1'', C–3''and C–4', H–3''→ C–1'', C–2'', C–4''and C–5'', H_3_–4''→ C–2'', C–3'', and C–5'', H_3_–5''→ C–2'', C–3'', and C–4'', H–2'''→ C–1''', C–3''', and C–4''', H–3'''→ C–1''', C–2''', and C–4''', H–4'''→ C–2''', C–3''', C–5'''and C–6''', H_3_–5'''→ C–3''', C–4''', and C–6''', H_3_–6'''→ C–3''', C–4''', and C–5'''; (+)HRESIMS *m/z* 445.2914 [M + H]^+^ (Calculated for C_22_H_41_N_2_O_7_^+^, 445.2908).

**Table 1:** ^1^H and ^13^C NMR data of compound **1** in CD_3_OD^a^

No	1^a^	
	*δ*_C_ (carbon type)	*δ*_H_, multi (*J* in Hz)
1	178.1 (C)	–
2	71.6 (CH)	4.11 dd (10.0, 3.5)
3	45.1 (CH_2_)	1.56 ddd (13.6, 9.3, 3.5) 1.51 ddd (13.6, 10.0, 4.8)
4	25.7 (CH)	1.87 m
5	24.1 (CH_3_)	0.96 d (6.6)
6	21.9 (CH_3_)	0.96 d (6.5)
1′	172.2 (C)	–
2′	58.9 (CH)	4.49 dd (8.7, 6.6)
3'	32.1 (CH)	2.27 m
4'	19.8 (CH_3_)	0.98 d (6.8)
5'	18.5 (CH_3_)	0.97 d (6.8)
1''	171.1 (C)	–
2''	80.3 (CH)	4.90 d (5.2)
3''	32.0 (CH)	2.23 m
4''	19.4 (CH_3_)	1.00 d (7.0)
5''	17.9 (CH_3_)	0.98 d (6.8)
1'''	178.0 (C)	–
2'''	53.9 (CH)	4.39 brt (7.1)
3'''	42.9 (CH_2_)	1.64 dd (8.4, 7.1)
4'''	24.8 (CH)	1.73 m
5'''	23.8 (CH_3_)	0.95 d (6.6)
6'''	22.2 (CH_3_)	0.93 d (6.6)

### Determination of absolute configurations at *α*-carbons in units using the* O*-Marfey’s method

Compound **1** (0.3 mg) was dissolved in 500 μL of 6 N HCl and stirred for 3 h at 121 ºC. After the hydrolysis of compound **1**, the product was separated into two equal portions for derivatization with either _L_-1-fluoro- 2,4-dinitrophenyl-5-leucine amide (FDLA) or _D_-FDLA. Each portion was dissolved in 1 mL of tetrahydrofuran with 2 mg of sodium hydride. Finally, 1 mg of _L_-FDLA or _D_-FDLA was added and the mixture was stirred under argon for 5 min at 27 ºC. Then, a 30 μL aliquot of the reaction mixture was removed and quenched by adding 10 μL of 2 N HCl. LC–MS analysis was performed using a Phenomenex reverse-phase C_18_ column (150 mm × 4.6 mm) at a flow rate of 0.4 mL/min. The linear gradient elution from 10 to 100% aqueous CH_3_CN (0.1% FA) for 30 min was used as the mobile phase and ESI was used as the ionization method. Two peaks referred to as the _L_- and _D_-FDLA derivatives of Leu were observed at 18.54 and 21.40 min (*m/z* 426.4, [M + H]^+^), respectively. Two peaks for the _L_- and _D_-FDLA derivatives of leucic acid were observed at 21.36 and 18.54 min (*m/z* 427.4, [M + H]^+^), respectively. Two peaks for the _L_- and _D_-FDLA derivatives of Val were observed at 19.95 and 17.37 min (*m/z* 412.4, [M + H]^+^), respectively. Two peaks for the _L_- and _D_-FDLA derivatives of hydroxyisovalerate (Hiv) were observed at 17.25 and 19.87 min (*m/z* 413.4, [M + H]^+^), respectively.

### Bioassays

Antibacterial activity was evaluated against *E*. *coli* DH10B and *Pseudomonas putida* ATCC17453 strains at 1 μg scale of compound **1**, following the procedure from previous reports (Meca et al. 2010). Antibodies against iNOS (#13120), COX-2 (#12282), and GAPDH (#2118) were purchased from Cell Signaling Technology (Danvers, MA, USA). The RAW 264.7 murine macrophage cells were purchased from the Korea Cell Line Bank (Seoul, Korea) and cultured in Dulbecco’s modified eagle medium (DMEM) (Corning, Corning, NY, USA) containing 10% fetal bovine serum (ATCC, Manassas, VA, USA) and 1% penicillin/streptomycin (Gibco, Grand Island, NY, USA) at 37 ℃ and 5% CO_2_ conditions. Cell viability was measured using the EZ-Cytox assay which was optimized for small amounts of compound in our previous study (Um et al. 2023). RAW264.7 cells were seeded onto a 96-well microplate at 0.5 × 10^5^ cells/well and cultured for 24 h. The cells were pretreated with compound **1** at concentrations of 0.8, 1.6, 3.1, 6.3, 12.5, 25, 50, 75, and 100 μmol/L (diluted in DMEM) for 24 h. The following day, the EZ-Cytox (DoGENBio, Seoul, Korea) solution was added to each well and allowed to react for 30 min. Cell viability was measured using a microplate reader (Molecular Devices, San Jose, CA, USA) at 450 nm. Medium containing no sample and dexamethasone (Invivogen, San Diego, CA, USA) at 100 umol/L were used as negative and positive controls, respectively.

RAW264.7 cells were inoculated onto a 96-well microplate at 0.5 × 10^5^ cells/well and cultured for 22 h. Cells were pretreated with compound **1** at concentrations of 0.8, 1.6, 3.1, 6.3, 12.5, 25, 50, 75, and 100 μmol/L for 2 h and induced for an inflammatory response using LPS (500 ng/mL) from *E. coli* 0111:B4 (Sigma-Aldrich, St. Louis, MO, USA) (500 ng/mL) for 22 h. The collection of culture media for the detection of nitric oxide (NO) was performed with Griess reagent (Sigma-Aldrich, St. Louis, MO, USA) using a microplate reader at a wavelength of 550 nm in the dark for 10 min.

All values are presented as means ± standard deviation (SD). Statistical comparisons were performed using one-way analysis of variance and Tukey’s post hoc test. Significance levels were defined as P-values, with ***p < 0.0001, **p < 0.001, and *p < 0.05.

### Immunoblotting

RAW 264.7 cells were inoculated onto a six-well plate (0.5 × 10^6^ cells/well) and cultured at 37 °C in a 5% CO_2_ incubator overnight. The following day, the cells were pretreated with compound **1** (50 and 100 μmol/L) for 2 h and stimulated with LPS (500 ng/mL) for 22 h. Proteins were harvested using radioimmunoprecipitation assay buffer, separated on polyacrylamide gels, and transferred to polyvinylidene fluoride membranes. These membranes were blocked with 5% skim milk in tris-buffered saline with tween 20 (tween 20%; 0.001%) and incubated with primary antibodies at 4 °C overnight. The membranes were then incubated with horseradish peroxidase-conjugated secondary antibodies at room temperature for 1 h. The immunoreactivity was detected using ECL (GE Healthcare Biosciences, Waltham, MA, USA). Protein band intensities were quantified using the ImageJ software (NIH, Bethesda, MD, USA). The GAPDH antibody was used as an internal control. Protein quantities were normalized to the intensity of internal control bands.

## Results

### Analysis of biosynthetic gene cluster and heterologous expression of SNJ102 BGC 24

**Fig. 1:**
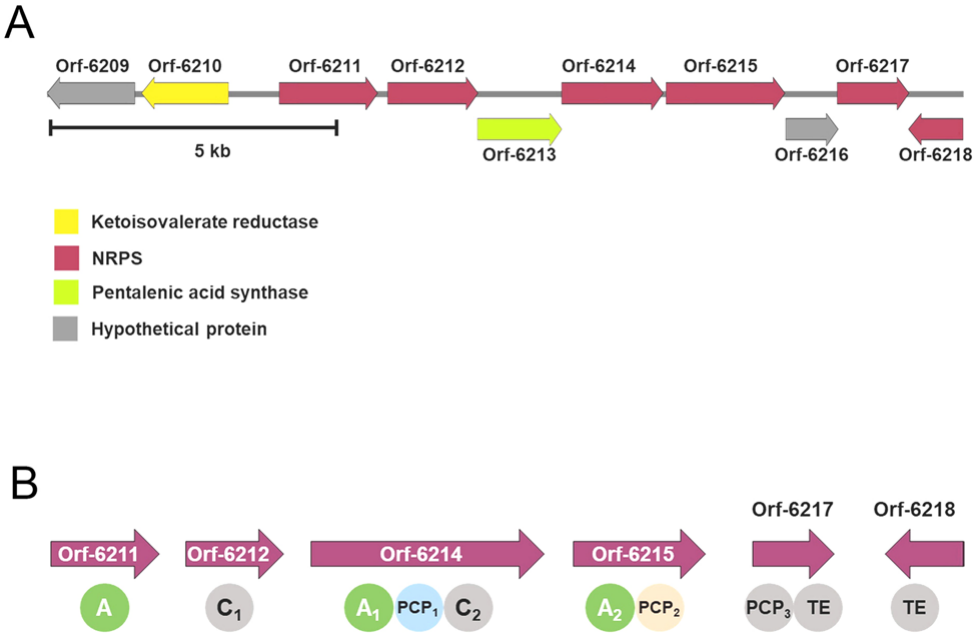
**A** Biosynthetic gene cluster of SNJ102 BGC 24 and **B** domain organization of the NRPS genes predicted by antiSMASH

Analysis of the secondary metabolite BGCs of the SNJ102 strain using antiSMASH5.0 (Blin et al. 2019) predicted 31 putative BGCs: five polyketide (PK)-NRP hybrids, five terpenes, four PK, three NRP, three bacteriocins, three siderophores, two lanthipeptides, two melanins, one ectoine, one indole, one phenazine, and one butyrolactone BGCs. Among them, two NRP, one PK, and one PK-NRP hybrid BGCs appeared nonfunctional because of the presence of only a few single domains in their BGCs. An in-depth analysis of the domains and modules of the three predicted NRPSs revealed a noncanonical NRPS, SNJ102 BGC 24 (BGC 24), positioned between 6,957,310 and 6,971,565 bp on the linear chromosome of SNJ102. Unlike the canonical NRPS, BGC 24 contained incomplete monodomain and didomain modules (Fig. 1). Protein BLAST analysis of the genes predicted that they resembled various depsipeptide-producing NRPSs (Table S3). Probably due to the noncanonical features of BGC 24, antiSMASH5.0, was not able to predict the amino acid or hydroxycarboxylic acid substrates of the A domains in BGC 24 NRPS. To further investigate BGC 24, it was captured using transformation-associated recombination (TAR) cloning and heterologously expressed in several *Streptomyces* host strains.

A BAC library consisting of 4224 BAC clones of the SNJ102 genome was first constructed and screened for a BAC clone containing BGC 24 via PCR amplification of BGC 24 NRPS modules (orf-6211, 6212, 6214, and 6215) (Fig. 1) and sequencing. Subsequently, a BAC clone carrying a 135 kb long SNJ102 genome fragment was selected (Fig. S1A). A genomic fragment containing BGC 24 was prepared from the BAC clone via enzymatic digestion, and the targeted gene cluster, BGC 24, was captured using the single-copy integration vector, pCB_Apr, through TAR cloning (Kim et al. 2023a) (Fig. S1B).

The selection of an appropriate *Streptomyces* host is crucial for the successful expression of a foreign gene cluster (Lee et al. 2019). For example, when tunicamycin B2, griseorhodin A, and cinnamycin BGCs were introduced into *S. albus* J1074, Del14, B2P1, B4 and *Streptomyces coelicolor* M1152 and M1154, only four *S. albus* strains were found capable of producing tunicamycin B2 and griseorhodin A. Cinnamycin was biosynthesized in all strains; however, the production titer was significantly greater in *S. coelicolor* M1152 for reasons that remain unidentified (Myronovskyi et al. 2018). The plasmid carrying BGC 24, named pCB_102DP, was introduced into three different *Streptomyces* heterologous host strains, *S. albus* J1074, *S. venezuelae* YJ028, and *S. roseosporus* NRRL 11379, via conjugation, and integrated into each genome using ΦC31 integrase. Gene expression of BGC 24 was initially analyzed in three *Streptomyces* strains to select a suitable host strain. Each strain with BGC 24 was cultured in R5^−^, ISP2, and TSBY media. Regardless of the medium used for bacterial cultures, *S. roseosporus* failed to express any genes; whereas, *S. venezuelae* expressed a portion of the BGC 24. *S. albus* was the only strain capable of expressing all of the BGC 24 biosynthetic core genes in every growth medium that was examined (Fig. S2). LC–MS analysis of crude extracts from the cultures of the control, *S. albus*::pCB_Apr, and *S. albus*::pCB_102DP confirmed the production of a new compound (Fig. S3A). To elucidate the structure of the new compound produced by BGC 24, 0.7 mg of compound **1** was isolated from a total of 30 L *S. albus*::pCB_102DP cultures in R5^−^ media.

### Structure elucidation of the compound 1

**Fig. 2:**
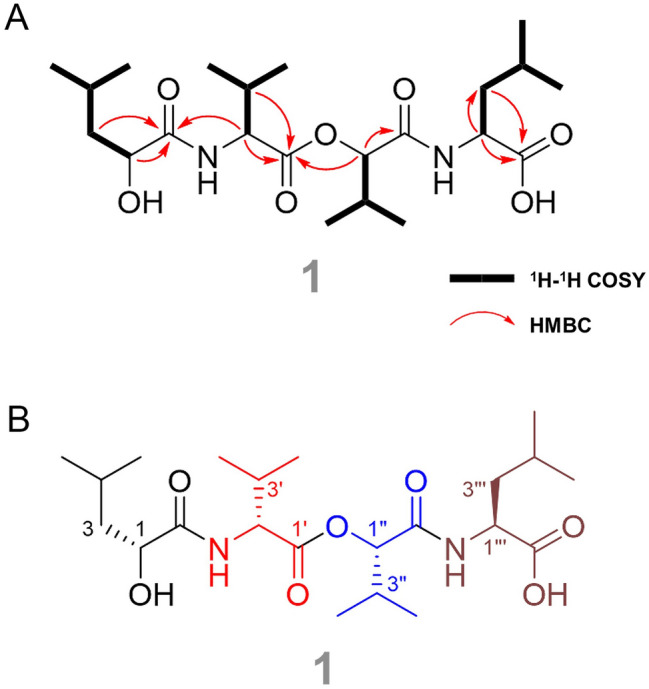
**A** Key ^1^H–^1^H COSY (bold lines) and HMBC (red arrows) correlations for compound **1**. **B** Complete structure of compound **1** showing the stereochemistry

**Fig. 3:**
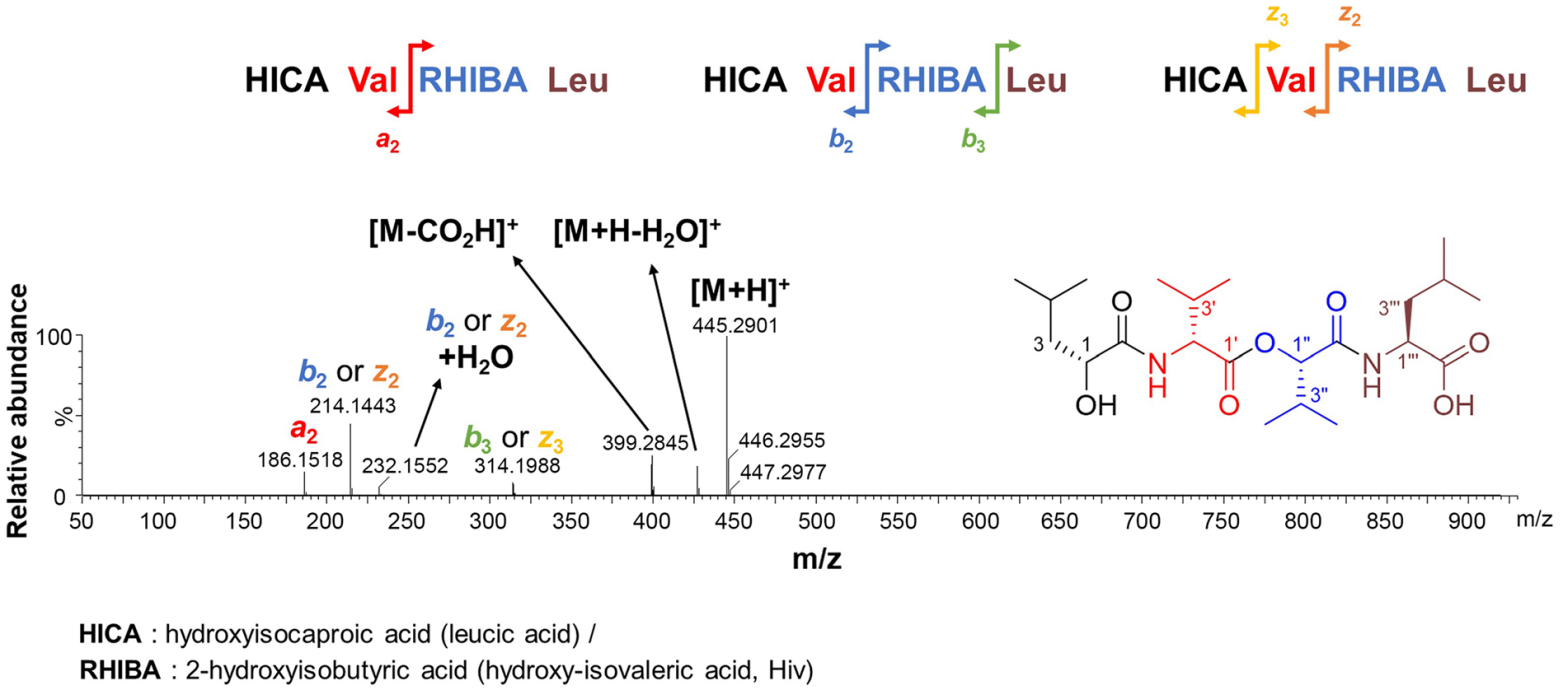
Chromatogram of LC–MS/MS fragmentation of compound **1**. *a*, *b*, and *z* fragments of the depsipeptide is indicated by arrows on the depsipeptide sequence

Compound **1** was isolated as a colorless gel. Its molecular formula was established as C_22_H_40_N_2_O_7_ (four unsaturated) using (+) high-resolution electrospray ionization MS (observed at *m/z* 445.2914 [M + H]^+^, calculated for C_22_H_41_N_2_O_7_^+^, 445.2908), and its UV absorption was measured (Figs. S3B and S4). Analysis of ^1^H, ^13^C, and HSQC NMR data for compound **1** revealed that it had four carbonyl groups, two sp^3^ oxymethine groups, two nitrogen-bound methine groups, four non-oxygenated sp^3^ methines, two non-oxygenated methylenes, and eight methyl groups (Table 1 and Figs. S5–S7). As expected from the genomic study, compound **1** was a depsipeptide, as elucidated through the analysis of the 1D and 2D NMR spectra. The 1D NMR spectrum of compound **1** was much clearer when measured in CD_3_OD than when measured in CDCl_3_ and DMSO-*d*_6_. Based on the analysis of ^1^H, ^13^C, and HSQC NMR, two nitrogen-bound methine groups, C–2'/H–2'(*δ*_C_ 58.9, *δ*_H_ 4.49) and C–2'''/H–2'''(*δ*_C_ 53.9, *δ*_H_ 4.39), were identified; these groups corresponded to the *α* positions of two amino acids. In addition, two methine groups were identified downfield, C–2/H–2 (*δ*_C_ 71.6, *δ*_H_ 4.11) and C–2''/H–2''(*δ*_C_ 80.3, *δ*_H_ 4.90); these groups were oxygenated. The interpretation of ^1^H-^1^H COSY spectrum showed four separate spin systems, two CH–CH_2_–CH(CH_3_)–CH_3_ and two CH–CH(CH_3_)–CH_3_, indicating the presence of leucine (Leu), leucic acid, valine (Val), and Hiv (Fig. S8). HMBC correlations of *α*- and *β*-protons with carbonyl carbons in each amino acid demonstrated that they belonged to two amino acids and two hydroxy-substituted amino acids (Figs. 2A and S9). The carbonyl carbons C–1 (*δ*_C_ 178.1) and C–1'''(*δ*_C_ 178.0) of terminal amino acids, leucic acid and leucine, could be assigned by the HMBC correlations of H–2 (*δ*_H_ 4.11) and H–3 (*δ*_H_ 1.56, 1.51) with C–1 (*δ*_C_ 178.1) and of H–2'''(*δ*_H_ 4.39) and H–3'''(*δ*_H_ 1.64) with C–1''', respectively. And the HMBC correlations of H–2'(*δ*_H_ 4.49) and H–3'(*δ*_H_ 2.27) with the carbonyl carbon at *δ*_C_ 172.2 confirmed the assignment of the carbon to be C–1'of Val. And the last carbonyl carbon at *δ*_C_ 171.1 was confirmed to be C–1''of Val by the HMBC correlations of H–2''and H–3''with C–1''. The HMBC correlation of the nitrogen-bound methine H–2'with C–1 (*δ*_C_ 178.1) suggested that leucic acid was adjacent to Val. Then, the HMBC correlation of H–2''with C–1'(*δ*_C_ 172.2) established the sequence from Val to Hiv. To confirm the connection between Hiv and Leu, MS/MS fragmentation was analyzed because the HMBC correlation of H–2'''with C–1''was not observed due to the limited amount of the compound; thus, high-resolution LC–MS analysis was performed to overcome this challenge. Because of the symmetry of the fragment ions of compound **1**, *b*_n_ and *z*_n_ ions had identical fragment masses. This made it difficult to distinguish *b* ions from *z* ions in the MS/MS fragmentation analysis. However, the presence of the *b*_3_ or *z*_3_ ion, an ion mass identical to [M–Leu]^+^ or [M–leucic acid]^+^, respectively, as well as the [M–CO_2_H]^+^ ion in the fragmentation of compound **1** confirmed that the terminal leucine was connected to Hiv (Figs. 2B and 3).

The absolute configuration of each chiral center in the depsipeptide was elucidated using the *O*-Marfey’s method with _L_-FDLA and _D_-FDLA following the acidic hydrolysis of compound **1** (Moon et al. 2013) (Fig. S10). The hydrolysate of compound **1** was derivatized with _L_-FDLA and _D_-FDLA and analyzed using LC–MS. Since the _L_-FDLA-Leu of the hydrosylate was eluted at an earlier retention time than _D_-FDLA-Leu of the hydrosylate, the absolute configuration of Leu was determined to be L (*R*_t_ of _L_-FDLA derivative: 18.54 min; *R*_t_ of _D_-FDLA derivative: 21.4 min). The _L_-FDLA-Hiv of the hydrosylate was also eluted earlier than its _D_-FDLA-Hiv, indicating L configuration of Hiv. In the case of leucic acid and Val, however, _L_-FDLA derivatives were eluted later than the _D_-FDLA derivatives, indicating D configuration of leucic acid and Val. Thus, the free amino acids in compound **1** were determined to be _L_-Leu, _D_-leucic acid, _D_-Val, and _L_-Hiv by comparing the elution order of the diastereomers. According to previous research, ketoisovalerate reductase (KIVR) isomerizes the configuration of stereogenic centers in amino acids during the conversion of amine groups to hydroxyl groups (Zhang et al. 2012). Thus, the dual function of KIVR could also explain the various chiralities in compound **1** (Fig. 2B), as a highly similar enzyme, orf- 6210, was identified in BGC 24.

### Anti-inflammatory effects of the compound 1

**Fig. 4:**
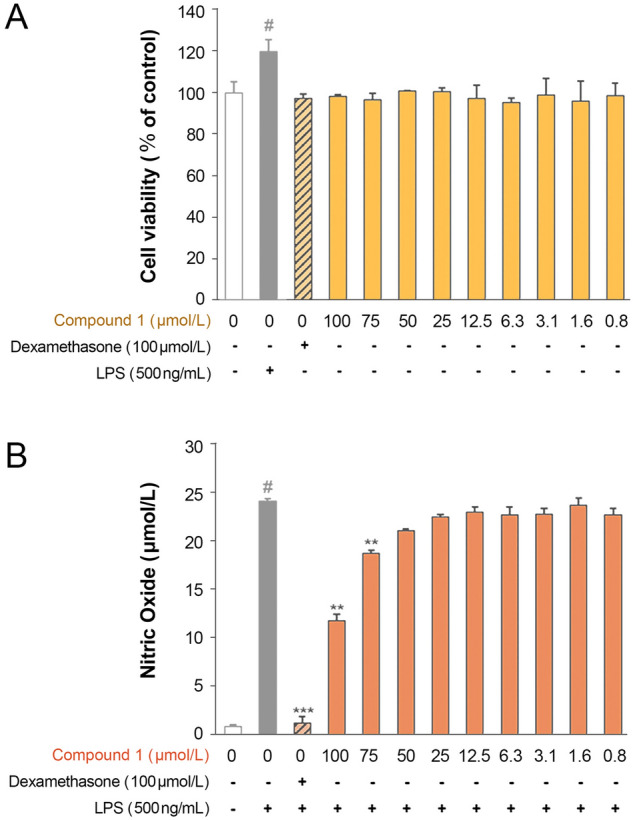
Effects of compound **1** on cytotoxicity and nitric oxide production in LPS-stimulated RAW264.7 cells. **A** RAW264.7 cells were treated with compound **1** for 24 h. **B** RAW264.7 cells were treated with compound **1** at varying concentrations for 2 h and stimulated with LPS for 22 h. #*p* < 0.0001 vs. the control group, ***p < 0.0001 vs. the LPS group (*n* = 2)

Beauvericin and bassianolide, two fungal depsipeptides that share structural similarities with compound **1**, exhibit antibacterial activity against various Gram-negative microbes such as *E*. *coli* CECT 4782 and *Pseudomonas aeruginosa* CECT 4628 (Meca et al. 2010, Roig et al. 2014). The bioactivity of compound **1** was examined to determine whether it exhibited similar properties. Unlike the two fungal depsipeptides, compound **1** did not exhibit any bactericidal activity against *E*. *coli* DH10B and *P*. *putida* ATCC17453 strains, which might result from the linear configuration of compound **1**. However, compound **1** had anti-inflammatory effects in cells with LPS-induced inflammation. RAW264.7 cells were treated with compound **1** to evaluate the non-cytotoxic concentration range. Cell viability was assessed using the EZ-Cytox assay. Treatment of 500 ng/mL LPS in the RAW264.7 cells increased the cell viability by 119.9 ± 5.7%. Treatment of compound **1** at concentrations of 100, 75, 50, 25, 12.5, 6.3, 3.1, 1.6, and 0.8 µmol/L increased the cell viability by 97.2 ± 2.1, 98.2 ± 0.7, 96.6 ± 3.1, 101.0 ± 10.3, 100.7 ± 1.6, 97.1 ± 6.6, 95.2 ± 2.1, 98.9 ± 7.9, 96.0 ± 9.8 and 98.5 ± 6.1%, respectively, indicating that compound **1** did not affect the cell viability at the concentrations investigated (Fig. 4A). Next, NO production was measured in the groups treated with LPS, LPS-dexamethasone, or LPS-compound **1**. NO levels of 0.9 ± 0.2, 24.1 ± 0.3, and 1.3 ± 0.7 µmol/L were produced from the control, LPS-treated, and LPS-dexamethasone treated RAW264.7 groups, respectively. The LPS and compound **1** treatments at the same concentrations as that of the cell viability test resulted in NO production of 11.8 ± 0.7, 18.8 ± 0, 21.1 ± 0.2, 22.5 ± 0.3, 23.1 ± 0.5, 22.8 ± 0.8, 22.8 ± 0.6, 23.7 ± 0.7 and 22.8 ± 0.7 µmol/L, respectively (Fig. 4B). A decrease in NO production, reduction by 2.5-fold, was observed in the 100 µmol/L compound **1**-treated RAW264.7 group. Subsequent experiments, evaluating the expression of COX- 2 and iNOS protein, were performed using 100 µmol/L of compound **1**.

**Fig. 5:**
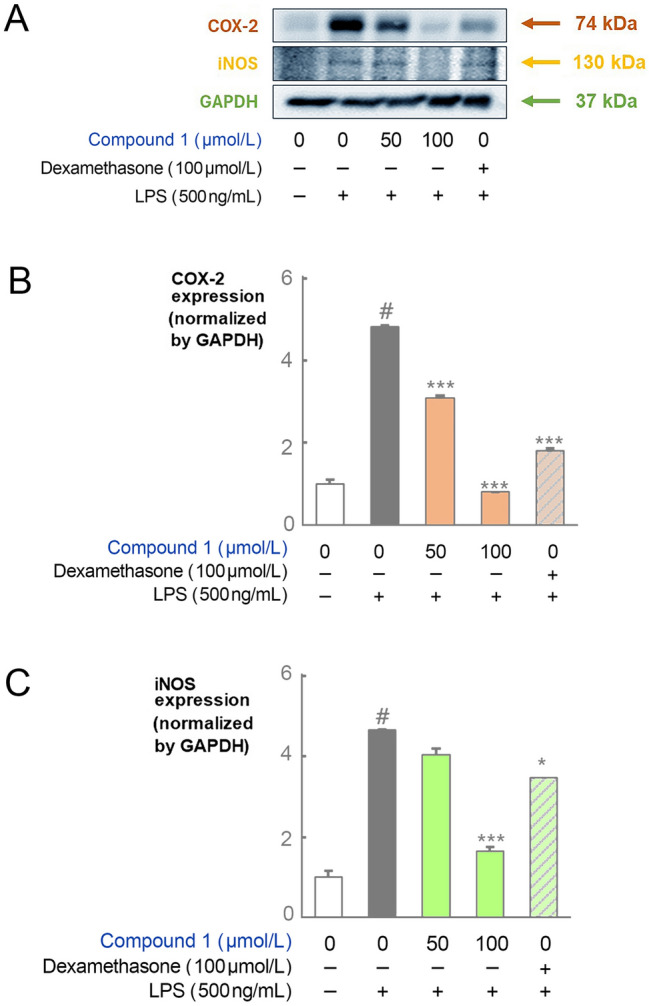
**A** Effect of compound **1** on iNOS and COX-2 protein expression in LPS-stimulated RAW264.7 cells. RAW264.7 macrophages were treated with compound **1** for 2 h and stimulated with LPS for 22 h. The control group was treated with 0.05% dimethyl sulfoxide. **B** COX-2 expression and **C** iNOS expression levels were normalized via GAPDH expression. The data are presented as the mean ± standard deviation (SD) of three independent experiments. #*p* < 0.0001 vs. the control group, ***p < 0.0001 or *p < 0.05 vs. the LPS group (*n* = 2)

The expression of COX-2 increased 4.8- and 1.8-fold in LPS- and LPS-dexamethasone treated RAW264.7 groups, respectively. Supplementation with 50 or 100 µmol/L compound **1** to LPS-treated groups resulted in 3.1- and 0.8-fold changes in COX-2 expression, respectively (Fig. 5A, B). This suggests that compound **1** treatment at higher concentrations inhibited the LPS-induced expression of COX-2. iNOS expression increased by 4.7- and 3.5-fold in the LPS- and LPS-dexamethasone-treated groups, respectively. In the LPS treated samples, compound **1** supplementation at 50 µmol/L did not alter iNOS expression; however, at 100 µmol/L, iNOS expression was repressed by 3.1-fold (Fig. 5A, C). Therefore, compound **1** at 100 µmol/L also inhibits LPS-induced iNOS expression.

## Discussion

Conventional gene inactivation for the study of biosynthesis is unfeasible, as iterative biosynthesis may lead to potential abolishment of product or intermediate biosynthesis. The investigation of the biosynthesis of compound **1** requires comprehensive analysis including interactions between NRPS modules and individual domains. Therefore, we proposed the biosynthetic pathway based on fungal depsipeptide biosynthetic models. Several biosynthetic pathways involving fungal depsipeptides have been proposed. Among them, two biosynthetic models have been proposed for iterative depsipeptides in fungi, whose structure resembles that of compound **1**. Further studies are required to determine their biosynthetic mechanisms (Alonzo et al. 2020, Steiniger et al. 2017). Nevertheless, the biosynthesis of compound **1** can be hypothesized using two well-accepted iterative fungal depsipeptide biosynthesis models: parallel and looping, with the parallel model carrying out the conventional sequential elongation of depsipeptide chains, and the looping model performing the back-and-forth condensation and esterification on the PCP domains (Fig. S11 A).

### Prediction of jejumide biosynthesis using the parallel model

The NRPS (BGC 24) synthesizing compound **1**, jejumide, resembles beauvericin (Meca et al. 2010) (Fig. S11B). Except for the presence of the extra stand-alone A domain and terminal TE domain in BGC 24 as well as a methyl transferase domain between the C_2_ and PCP_2_ domains in the beauvericin NRPS, the organization of protein subunits, the composition and order of NRPS domains are largely similar to each other (Fig. S11 C).

**Fig. 6:**
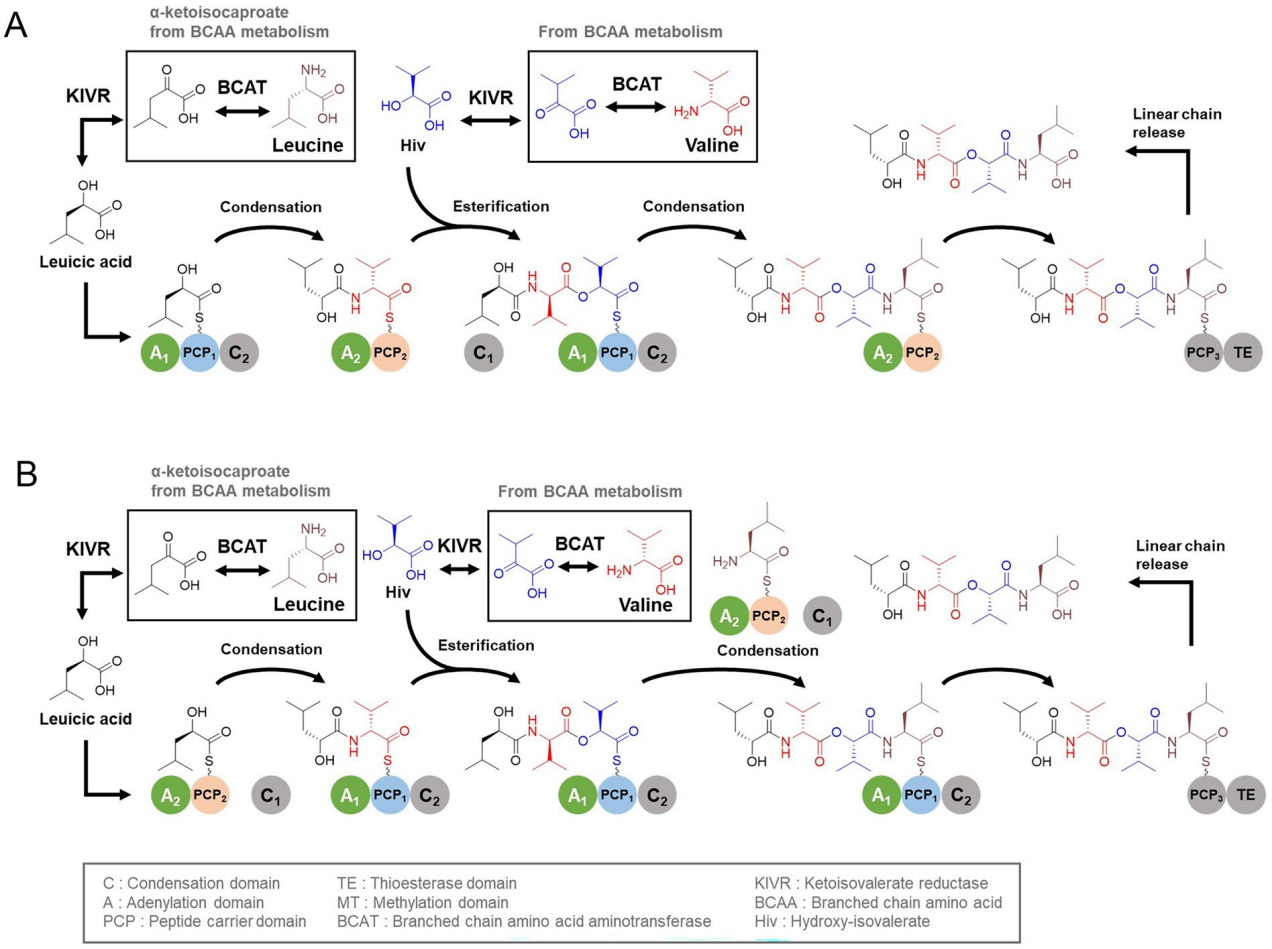
**A** Proposed parallel model and **B** looping models of compound **1** biosynthesis based on the proposed parallel and looping fungal depsipeptide biosynthesis models, respectively

In the parallel model, depsipeptide chain elongation occurs sequentially (Fig. 6A). Each module is responsible for one type of reaction at a time: esterification by the C_1_ single domain on the A-PCP_1_-C_2_ module and condensation by the C_2_ domain on A_2_-PCP_2_ di-domain module. The primary substrates for the biosynthesis of compound **1** probably originate from metabolism of branched chain amino acids of the host strain. The α-ketoisocaproate, produced directly from Leu by branched chain amino acid aminotransferase (BCAT) of the heterologous host, is first converted into leucic acid by KIVR. Leucic acid and Val are activated by the A_1_ and A_2_ domains and loaded onto the PCP_1_ and PCP_2_ domains, respectively. Peptidyl bonds between the two substrates are formed on PCP_2_ by the C_2_ domain, followed by the esterification with Hiv by the C_1_ domain and chain transfer to PCP_1_. Hiv is synthesized through the conversion of valine by BCAT and KIVR and subsequently activated by the A_1_ domain. Hiv is then loaded onto PCP_1_ prior to esterification. The last condensation reaction with Leu, which is activated by the A_2_ domain and loaded onto another PCP_2_, completes the depsipeptidyl intermediate formation. During fungal depsipeptide biosynthesis, it is predicted that the intermediate is transferred from the PCP_2_ domain to the PCP_3_ domain and is then released from the NRPS through the terminal C domain (C_3_ domain in beauvericin NRPS, Fig. S11C). The transfer of the SNJ102 depsipeptide intermediate from the PCP_2_ to PCP_3_ domain would occur similarly; however, compound **1** would be released from the NRPS through the terminal TE domain. The alignment of BGC 24 C_1_ and C_2_ domains with other fungal cyclic depsipeptides showed that the two C domains have homology with the typical NRPS C domain (Fig. S12). The C_1_ and C_2_ domains of bassianolide, beauvericin, and enniatin exhibited conserved active site motifs of HXXVDS and HHIXXDGWS, respectively. The C_T_ (terminal C) domains, also annotated as C_3_, of bassianolide, beauvericin, and enniatin contain a conserved active site motif of SHALYDGLS. The active site residues of BGC 24 C_1_ and C_2_ domains, HXXVDS and HHIXXDGWS, respectively, signified their homology with the C_1_ and C_2_ domains of fungal depsipeptide biosynthesis. Comprehensive further studies are necessary to identify the differences between the C domains responsible for conventional condensation and esterification. Phylogenetic analysis of BGC 24 C domains indicated a general divergence from fungal C domains (Fig. S12), but active site residues of the BGC 24 C domains suggested that the TE domain of the PCP_3_-TE module of BGC 24 would be responsible for chain termination.

### Prediction of jejumide biosynthesis using the looping model

In the looping model (Fig. 6B), Val and Hiv are activated and loaded onto PCP_1_ via C_1_ and A_1_, respectively. Similarly, Leu and leucic acid are activated and loaded onto PCP_2_ via C_2_ and A_2_, respectively. In contrast to the parallel model, in the looping model, condensation and esterification would occur in the PCP_1_ domain rather than back-and-forth transfer between PCP_1_ and PCP_2_, which could be a non-canonical characteristic predicted in the fungal depsipeptide biosynthesis model (Steiniger et al. 2017). The major difference between the proposed fungal looping model and that of BGC 24 may be the potential participation of the terminal C_3_ domain (Fig. S11C). The terminal C_3_ domain in the looping model is responsible for both esterification and chain release through macrocyclization (Yu et al. 2017); whereas, the C_3_ domain in the parallel model releases only the intermediate from the NRPS. However, the terminal PCP_3_-TE di-domain module of BGC 24 in the looping model would release compound **1**, just as it does in the aforementioned parallel model.

Macrocyclization may be the major difference between depsipeptides from fungi and *Streptomyces* sp. SNJ102. Fungal depsipeptides are released from the NRPS in a cyclic form, but compound **1** is released as a linear chain. Transcription of orf-6211, a stand-alone A domain, is confirmed (Fig. S2), but the translation is unidentified for now. The function of the stand-alone A domain remains unclear. Further extensive in vitro analysis of the interactions between NRPS modules and individual domains, utilizing purified NRPS proteins spanning from 50 to over 100 kDa with mass spectrometry analysis of the intermediate bound to the NRPS proteins, is required to elucidate the biosynthesis of compound **1**.

The terminal C domain determines the production of hexameric (six amino acids) and octameric (eight amino acids) fungal depsipeptides at different iterative stages of chain elongation and macrocyclization (Yu et al. 2017). Similar to those of the fungal depsipeptides, traces of dimeric depsipeptides corresponding to the octameric fungal depsipeptide were also discovered via high-resolution LC–MS analysis of the extracts from *S. albus*::pCB_102DP cultures (Fig. S13). Although only cyclic depsipeptides from fungal sources have been reported, the production of both linear and cyclic dimers of compound **1** is expected. Two compounds with masses identical to those of each dimeric form of compound **1** were further analyzed via LC–MS/MS fragmentation (Fig. S14). Differences of approximately 2 min between the retention times of the two putative dimers indicated that one may have a cyclic form. Owing to the symmetry in the structures, the LC–MS/MS fragmentation patterns of the two dimers were almost identical, with minor differences in the relative abundances of the fragment peaks. Because the production of dimeric forms of compound **1** was estimated to be hundreds of nanograms per 30 L of bacterial culture, further studies are required to isolate and distinguish the linear or cyclic forms of the two dimers.

### Suppression of iNOS and COX-2 gene expressions by jejumide

The inflammatory responses to NO and COX-2 are highly correlated with macrophage activation (Kim et al. 2023b, Palmer et al. 1988). NO has beneficial functions in the anticancer, antiviral, and inflammatory processes (Kleemann et al. 1993, Um et al. 2023). However, excessive NO production by iNOS, particularly macrophages, may stimulate inflammation and autoimmune disorders (Shan et al. 2009). Prostaglandin is a hormone-like molecule that initiates an inflammatory response and is biosynthesized from arachidonic acid by COX-2 (Fukata et al. 2008, Shan et al. 2009). NO is a well-known inducer of COX-2 expression. Excessive NO produced by iNOS stimulates COX-2 expression, leading to increased prostaglandin production and subsequent inflammation (Nogawa et al. 1998). We artificially stimulated inflammation in RAW264.7 cells by exposing them to LPS, which binds to Toll-like receptor 4 (TLR4) and upregulates COX-2 gene expression (Fukata et al. 2008, Shan et al. 2009, Surh et al. 2001). Supplementation of 100 µmol/L compound **1** inhibited the activation of iNOS and COX-2 expression caused by LPS in RAW264.7 cells and, thereby, reduced inflammation by managing the LPS-TLR4-induced gene expression signaling pathway.

## Conclusions

Genomic analysis of *Streptomyces* sp. SNJ102 revealed a non-canonical NRPS BGC that produces a new bacterial depsipeptide whose structure resembles that of a fungus-derived depsipeptide. In addition, jejumide exhibited anti-inflammatory effects, as evidenced by reduction of NO production and COX-2 expression in LPS-stimulated RAW264.7 cells. Further studies are necessary to understand the detailed biosynthetic mechanism of this intriguing noncanonical NRPS. The discovery of a fungal analog of the depsipeptide BGC in a genetically distant *Streptomyces* strain highlights the significance of exploring noncanonical NRPS from marine actinobacteria. Additionally, this study offers valuable insights into the development of novel synthetic biology tools and strategies for combinatorial biosynthesis that could be applied to the development of pharmaceuticals with enhanced bioactivity.

## Supplementary Information

Below is the link to the electronic supplementary material.

Supplementary file1 (DOCX 5716 KB)

## Data Availability

All data generated from this study are included in the supplementary information file.
