# Supplementary material for: Transformation-associated recombination and heterologous expression of noncanonical depsipeptide nonribosomal peptide synthetase derived from marine Streptomyces
Source: Mar Life Sci Technol. 2025 Apr 24;7(4):937–48. doi: 10.1007/s42995-025-00296-8 (PMC12662965; doi:10.1007/s42995-025-00296-8)
Supplement: Supplementary file 1 — Supplementary file1 (DOCX 5716 KB) [file 42995_2025_296_MOESM1_ESM.docx]

**Supporting Information**

**Transformation-Associated Recombination and Heterologous Expression of Noncanonical Depsipeptide Nonribosomal Peptide Synthetase Derived from Marine *Streptomyces***

Jeong Sang Yi^1^, Jin Won Choi^1^, Ngoc Han Le Thi^2^, Sung Jin Kim^2^, Hyun-Ju Kim^1^, Jung Min Kim^1^, Jun Eui Park^1^, Kyuho Moon^3^, Dong Chan Oh^1^, Sang Hee Shim^1^, Ki Sung Kang^2^*, Yeo Joon Yoon^1^*

^1^Natural Products Research Institute, College of Pharmacy, Seoul National University, Seoul 08826, Republic of Korea

^2^College of Korean Medicine, Gachon University, Seongnam 13120, Republic of Korea.

^3^College of Pharmacy, Kyung Hee University, Seoul 02447, Republic of Korea

Jeong Sang Yi, Jin Won Choi, and Ngoc Han Le Thi equally contributed to this work.

**Corresponding Author**

**Yeo Joon Yoon** – *Natural Products Research Institute, College of Pharmacy, Seoul National University, Seoul 08826, Republic of Korea*; orcid.org/0000-0002-3637-3103; Phone: +82 28802379; Email: [yeojoonyoon@snu.ac.kr](mailto:yeojoonyoon@snu.ac.kr)

**Ki Sung Kang** – *College of Korean Medicine, Gachon University, Seongnam 13120, Republic of Korea*; Phone: +82 317505402; Email: [kkang@gachon.ac.kr](mailto:kkang@gachon.ac.kr)

**Contents of Supporting Information**

**Table S1.** Primers used in this study

**Table S2.** Strains and plasmids used in this study

**Table S3.** Protein BLAST of genes in SNJ102 BGC 24

**Table S4.** Retention times of _L-_and _D-_ FDLA derivatives of the amino acids in compound **1**

**Fig. S1.** DNA gel image of the BGC capture template fragment and pCB_102DP plasmid map

**Fig. S2.** RT PCR of NRPS modules expressed in various heterologous host strains

**Fig. S3.** HRESIMS total ion chromatogram and analysis quality report of compound **1**

**Fig. S4.** UV spectrum of compound **1** (in MeOH)

**Fig. S5.** ^1^H NMR spectrum (800 MHz, CD_3_OD) of compound **1**

**Fig. S6.** ^13^C NMR spectrum (200 MHz, CD_3_OD) of compound **1**

**Fig. S7.** HSQC spectrum (800 MHz, CD_3_OD) of compound **1**

**Fig. S8.** ^1^H-^1^H COSY spectrum (800 MHz, CD_3_OD) of compound **1**

**Fig. S9**. HMBC spectrum (800 MHz, CD_3_OD) of compound **1**

**Fig. S10.** *O*-Marfey’s method analysis of compound **1**

**Fig. S11.** Comparison of depsipeptide BGCs

**Fig. S12.** Alignments of C domains from various depsipeptide BGCs

**Fig. S13.** Selected ion monitoring of crude extracts analyzed by qToF LC-MS

**Fig. S14.** MSMS fragmentation of dimeric depsipeptide

**References**

**Table S1.** Primers used in this study

| **Primer name** | **Sequence** | **Description** |
| --- | --- | --- |
| pCB_Apr_102_F | TCAGGACCCCGGCAAAGTGCCTGTATGTCGAAAGCTACATATAAGGA | Addition of SNJ102 depsipeptide BGC homology arm to the capture vector, pCB_Apr |
| pCB_Apr_102_R | GTCCATCGGTGCTGCCGAGGCCCCTACATAAGAACACCTTTGGTGGAG |  |
| 06211_F | TACGTCATGTACACCTCGGGATC | Plasmid screening and RT PCR of A-domain |
| 06211_R | CTTCCCAGAGAGATCACATCGG |  |
| 06212_F | AGACGATCGAGTTCTGGCTC | Plasmid screening and RT PCR of C-domain |
| 06212_R | GGTAGTTGAAGTAGCAGTCGAAC |  |
| 06214_F | GCCCCAGGACCTGCATGTAC | Plasmid screening and RT PCR of A-domain of A-PCP-C module |
| 06214_R | GGTCCGTAGCCGTTGGTCAG |  |
| 06215_F | TTCCTCCAAGAGCACGCGAT | Plasmid screening and RT PCR of A-domain of A-PCP module |
| 06215_R | GCCGTACCGAACTTCTCCTC |  |
| 06218_F | CTGTTCTGTTTCCCTTATGCGG | Plasmid screening and RT PCR of TE-domain |
| 06218_R | ACGACAGAAAGAAATAGCGTGAG |  |

**Table S2.** Strains and plasmids used in this study

| **Strain** | **Description** | **Reference** |
| --- | --- | --- |
| *E. coli* DH5α | Maintaining vectors | NEB |
| *E. coli* DH10B | Maintaining and manipulation of vectors and plasmids | NEB |
| *E. coli* ET12567 | Maintaining and manipulation of vectors and plasmids | Yi et al. 2020 |
| *Streptomyces* sp. SNJ102 | Native strain of depsipeptide BGC | This study |
| *S. serevisiae* BY4727 | Host strain for BGC capture | ATCC |
| *S. albus* J1074 | Heterologous expression host | Kim et al. 2023 |
| *S. venezuelae* YJ028 | Heterologous expression host | Han et al. 2011 |
| *S. roseosporus* NRRL11379 | Heterologous expression host | ATCC |
| **Vector** | **Description** | **Reference** |
| pESAC13A_102BAC | BAC clone containing SNJ102 depsipeptide BGC | This study |
| pCB_Apr | Integrative BGC capture vector with apramycin selection marker for bacteria and HIS3 and URA3 for yeast | Kim et al. 2023 |
| pRK2013 | Plasmid for triparental conjugation of plasmid DNA from *E. coli* to *Streptomyces* host strains | Jung et al. 2016 |
| pCB_102DP | pCB_Apr carrying SNJ102 depsipeptide BGC | This study |

**Table S3.** Protein BLAST of genes in SNJ102 BGC 24

| **Gene** | **Description** | **Gene Cluster of**  **Homologous Gene** | **Species** | **% ID** | **Blast Score** | **E-value** | **Reference** |
| --- | --- | --- | --- | --- | --- | --- | --- |
| orf06210 | Valine-pyruvate aminotransferase | KIVR, ketoisovalerate reductase | *Streptomyces fradiae* | 72 | 635 | 0.0 | Omura et al. 1983 |
| orf06211 | NRPS A  single domain | BE-43547A1, cyclic depsipeptide  Type I PKS hybrid | *Micromonospora* sp. RV43 | 44 | 307 | 5.13E-92 | Villadsen et al. 2016 |
| orf06212 | NRPS C  single domain | Hypeptin, cyclic depsipeptide | *Lysobacter* sp. K5869 | 26 | 56 | 8.34E-08 | Wirtz et al. 2021 |
| orf06214 | NRPS A-PCP-C domain module | Salinamide A, cyclic depsipeptide  Type I PKS hybrid | *Streptomyces* sp. CNB091 | 33 | 390 | 6.41E-114 | Hassan et al. 2015 |
| orf06215 | NRPS A-PCP  di-domain module | *ofaB*, orfamide A, cyclic depsipeptide | *Pseudomonas protegens* | 39 | 335 | 2.57E-100 | Jang et al. 2013 |
| orf06217 | NRPS PCP-TE  di-domain module | Skyllamycin A, cyclic depsipeptide | *Streptomyces* sp. Acta 2897 | 34 | 92 | 6.02E-20 | Schubert et al. 2014 |
| orf06218 | NRPS TE | Rakicidin D, cyclic depsipeptide | *Streptomyces lilacinus* | 39 | 173 | 5.22E-53 | Igarashi et al. 2010 |

**Table S4.** Retention times of _L-_and _D-_ FDLA derivatives of the amino acids in compound **1**

| **Amino acid** | **_L_-FDLA derivative** | **_D_-FDLA derivative** |
| --- | --- | --- |
| Leu | 18.54 | 21.4 |
| Leucic acid | 21.36 | 18.54 |
| Val | 19.95 | 17.37 |
| Hiv | 17.25 | 19.87 |

**
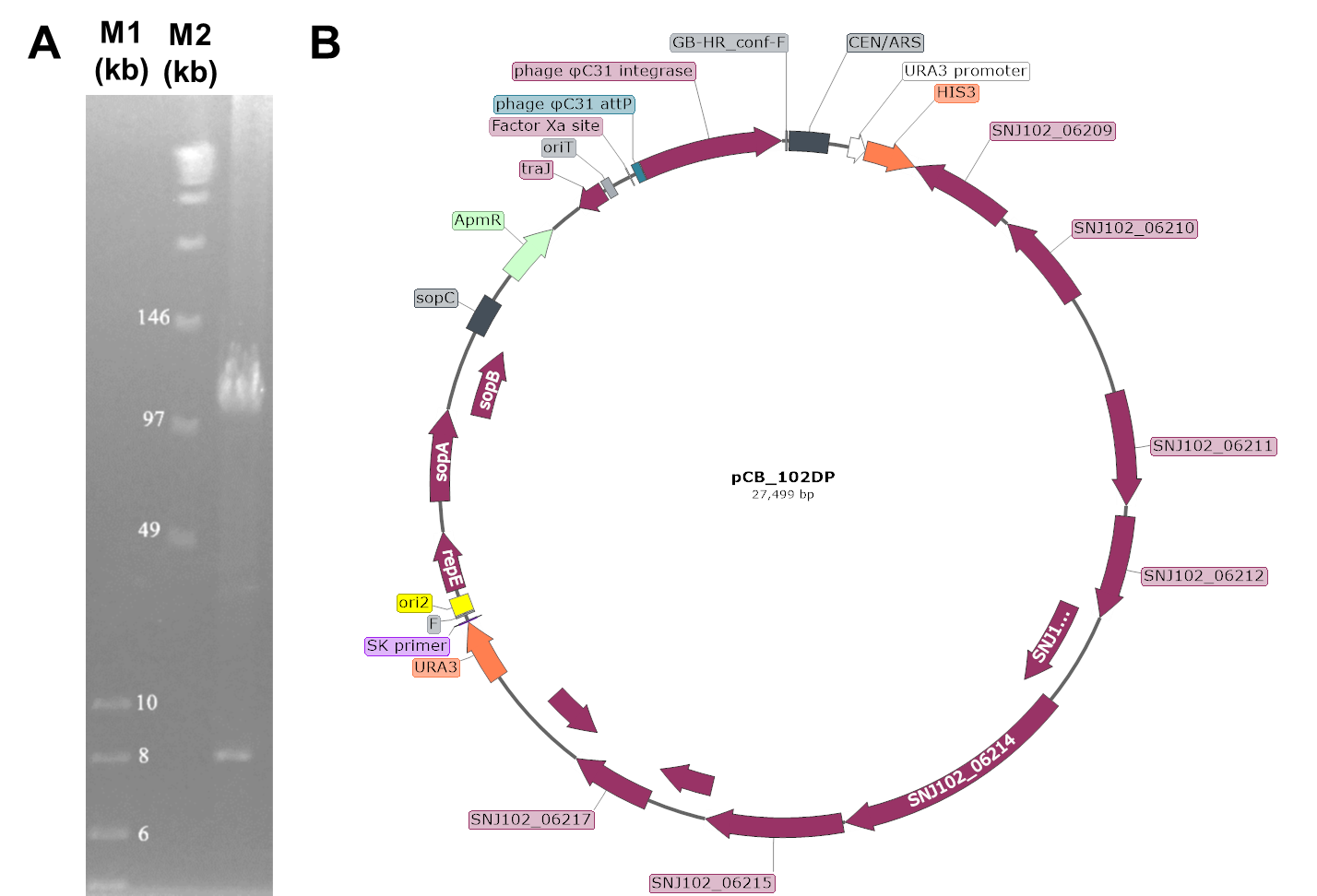
**

**Figure S1.** DNA gel image of the BGC capture template fragment and pCB_102DP plasmid map. **A** DNA gel image of *Dra*I digested SNJ102 BAC clone containing the target depsipeptide BGC. **B** A map of SNJ102 BGC 24 in pCB_Apr vector. SNJ102 BGC was inserted between the HIS3 and URA3 genes of pCB_Apr, replacing the pADH gene. A complementary sequence to that of SNJ102 BGC for the capture was inserted in primers by PCR amplification of the vector backbone.


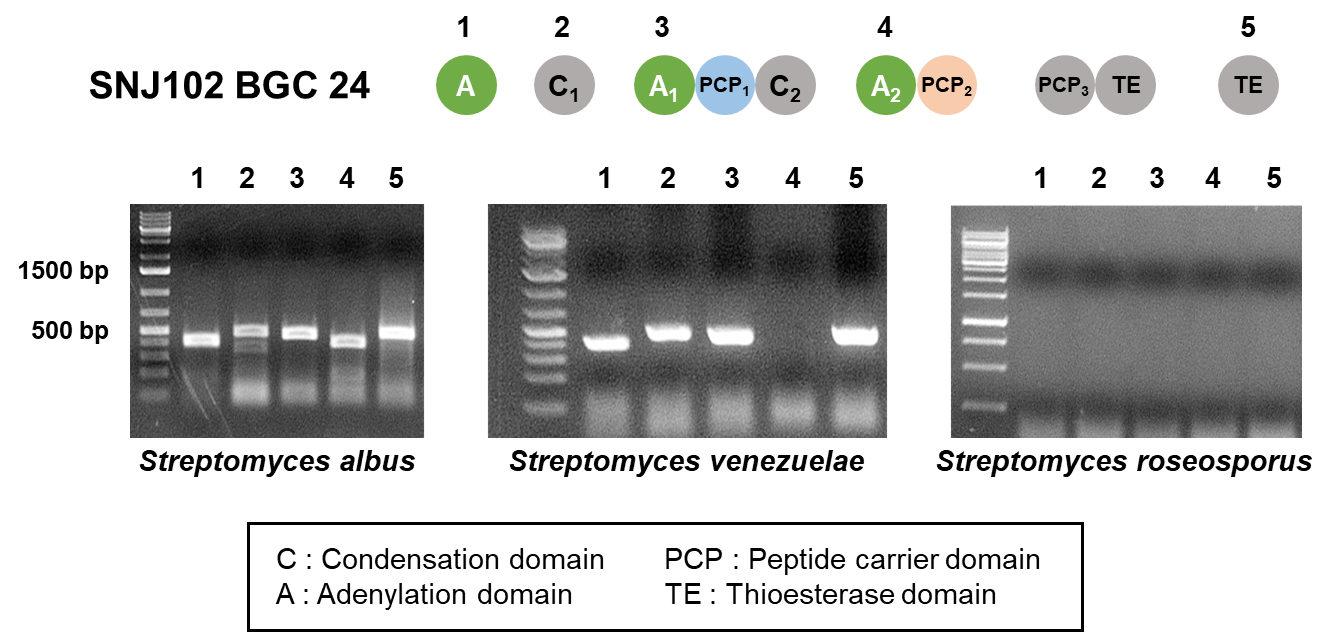


**Figure S2.** RT-PCR of NRPS modules expressed in various heterologous host strains cultured in R5^-^ medium. Depsipeptide BGC was heterologously expressed in *Streptomyces albus*, *Streptomyces venezuelae*, and *Streptomyces roseosporus*. mRNA was extracted, and cDNA was synthesized by reverse transcription.


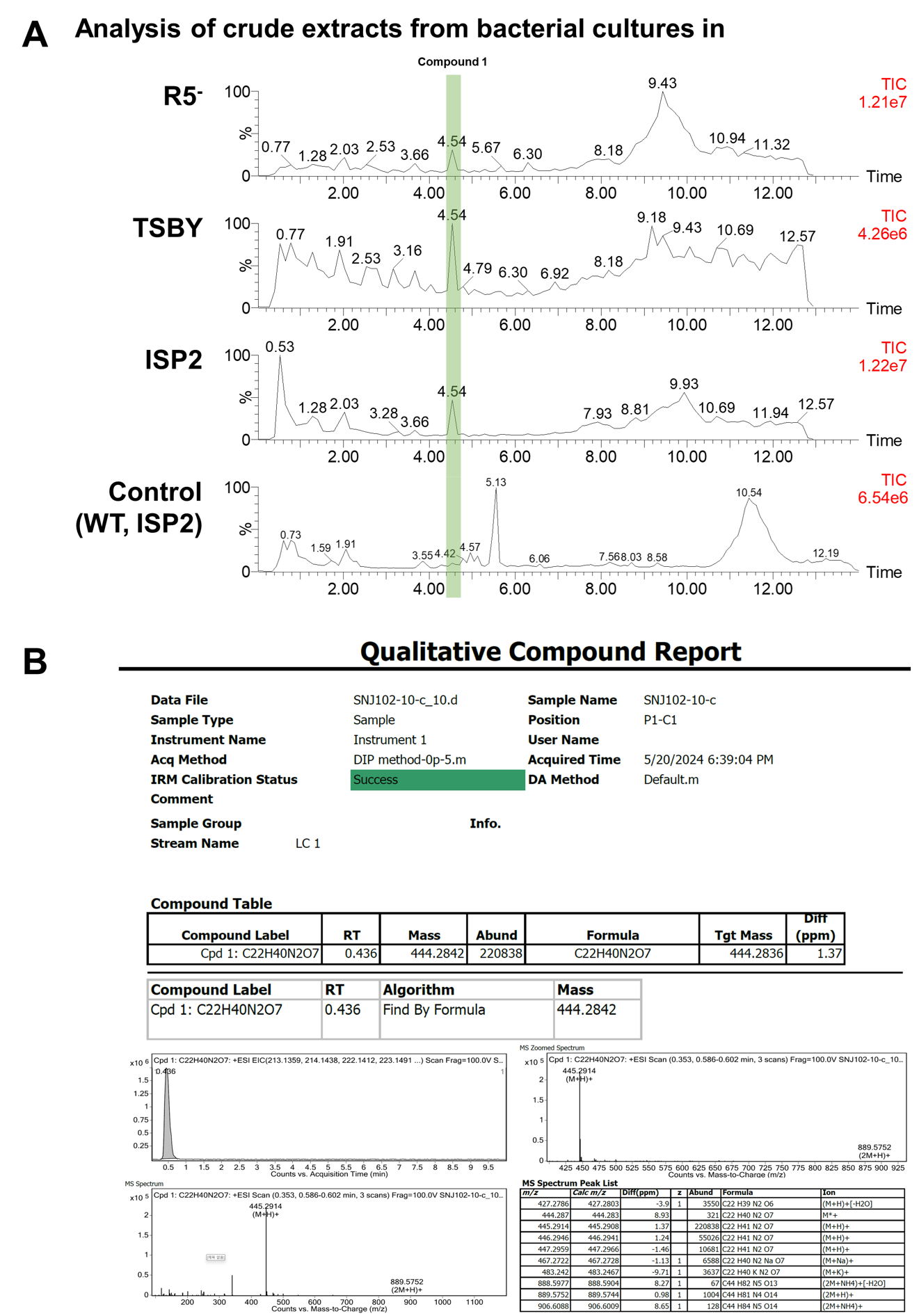


**Figure S3. A** HRESIMS total ion chromatogram and **B** HRESIMS analysis quality report of compound **1**


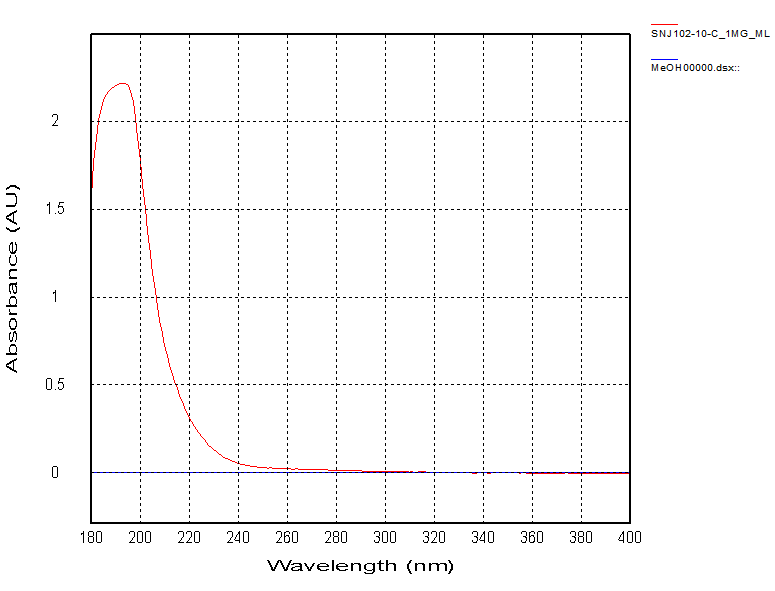


**Figure S4.** UV spectrum of compound **1** (in MeOH)

**
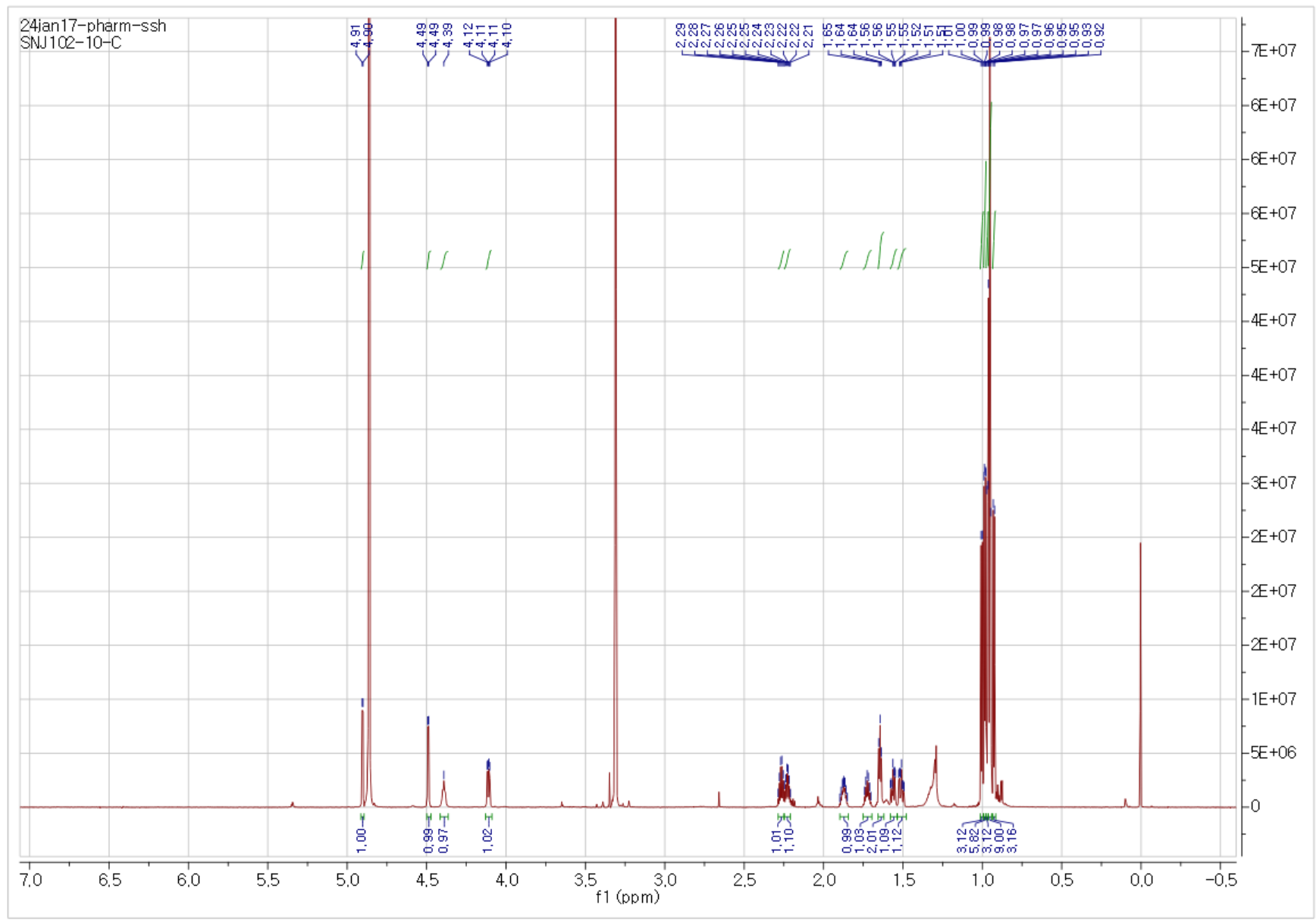
**

**Figure S5.** ^1^H NMR spectrum (800 MHz, CD_3_OD) of compound **1**


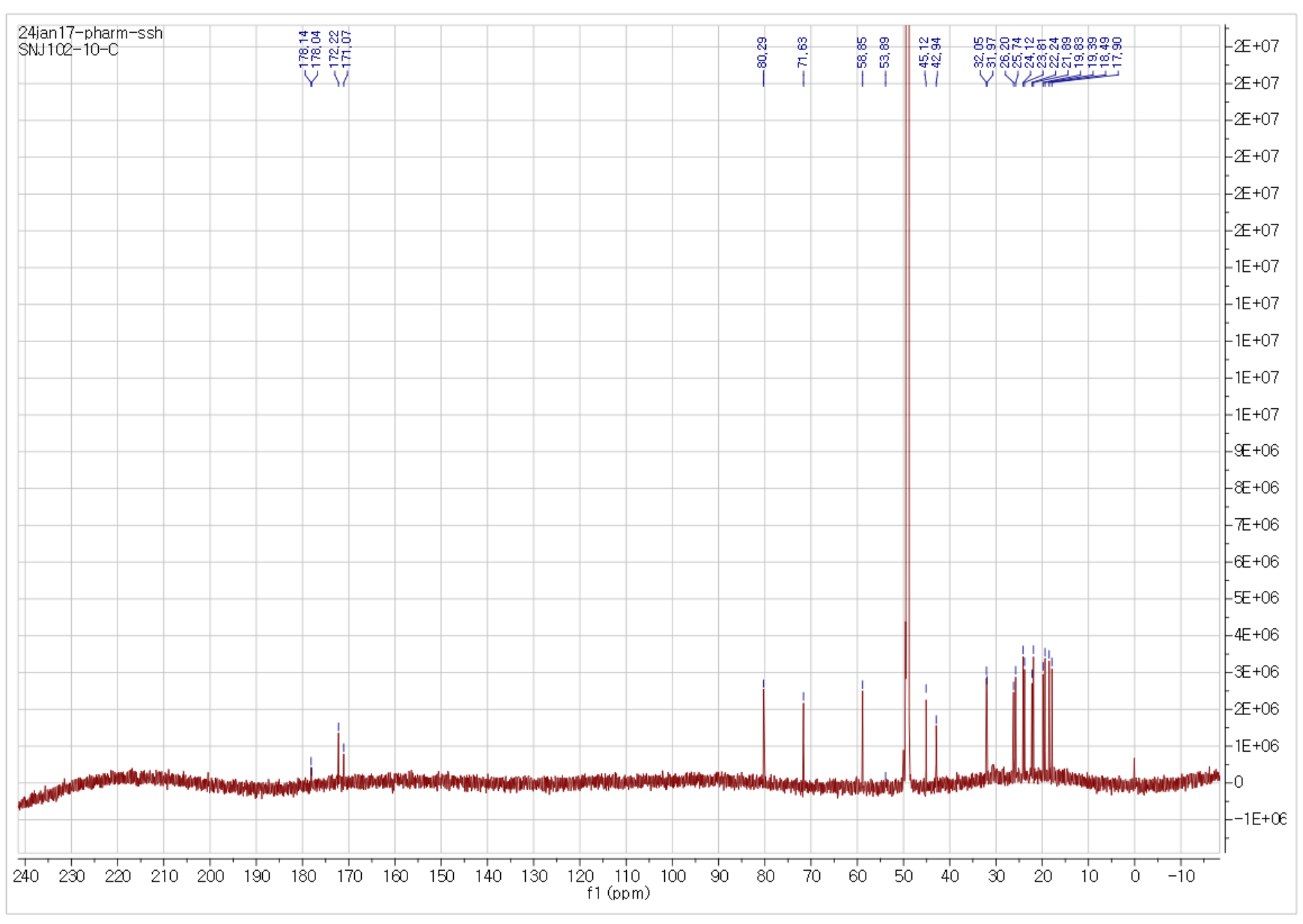


**Figure S6.** ^13^C NMR spectrum (200 MHz, CD_3_OD) of compound **1**

**
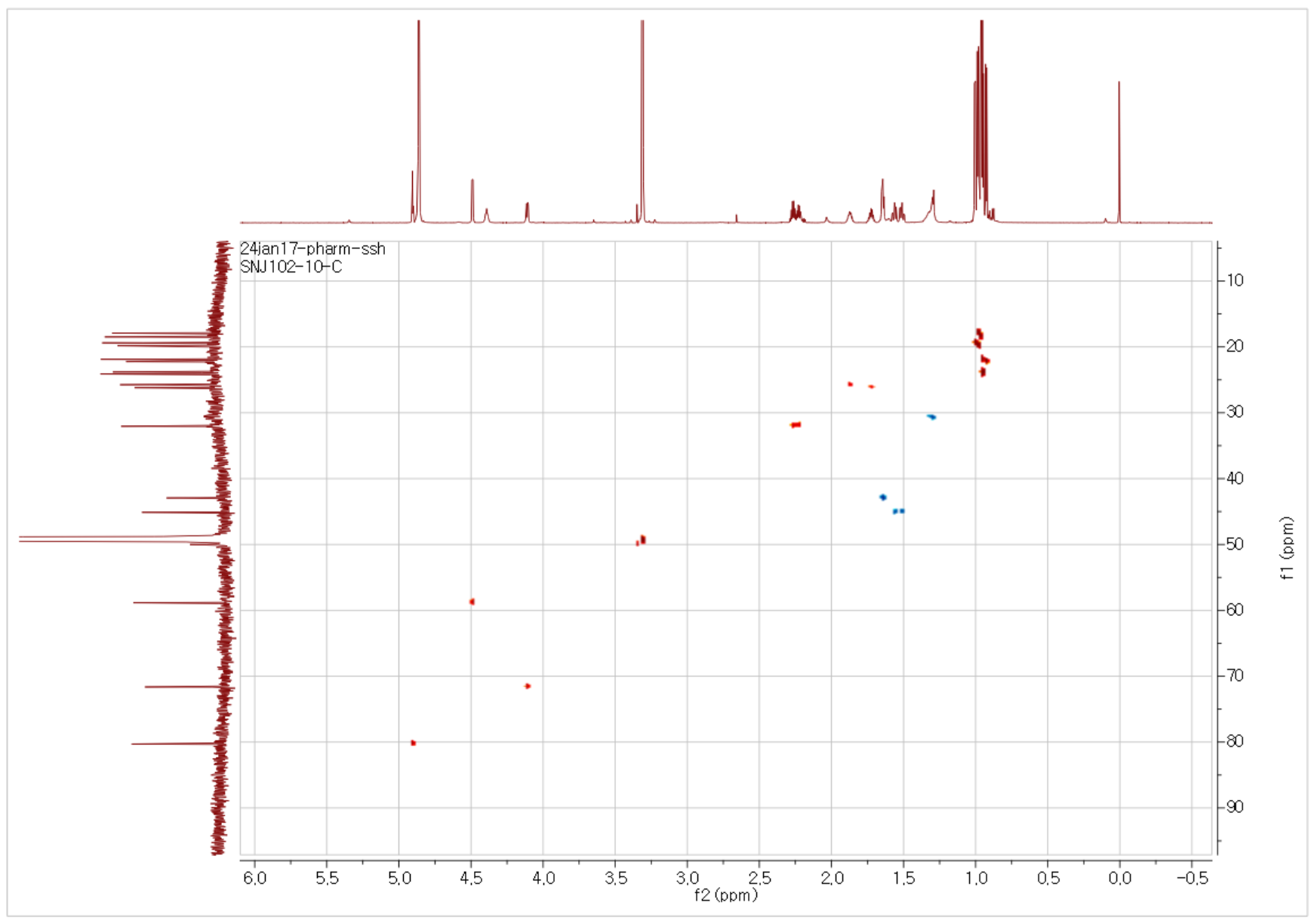
**

**Figure S7.** HSQC spectrum (800 MHz, CD_3_OD) of compound **1**


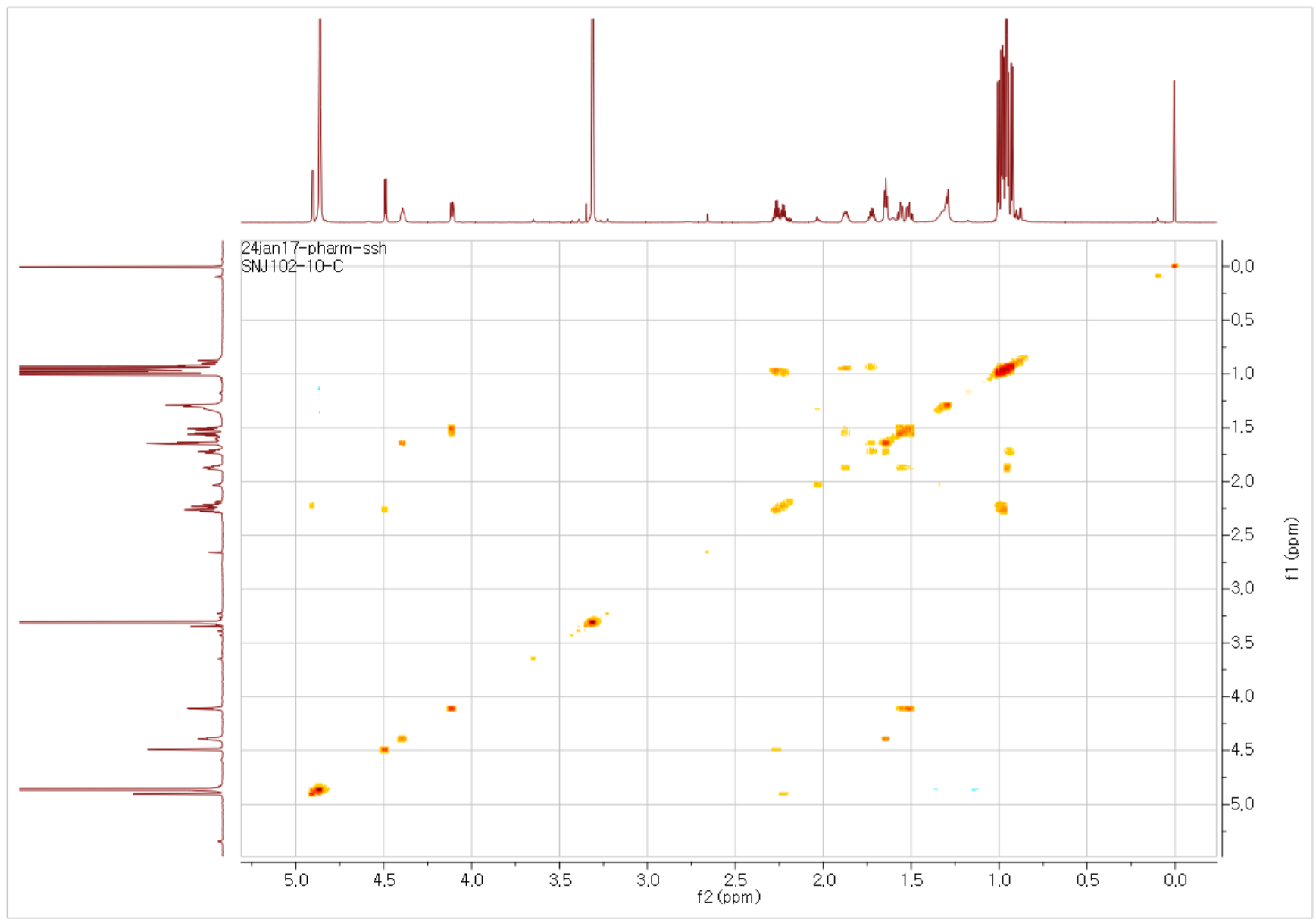


**Figure S8.** ^1^H-^1^H COSY spectrum (800 MHz, CD_3_OD) of compound **1**


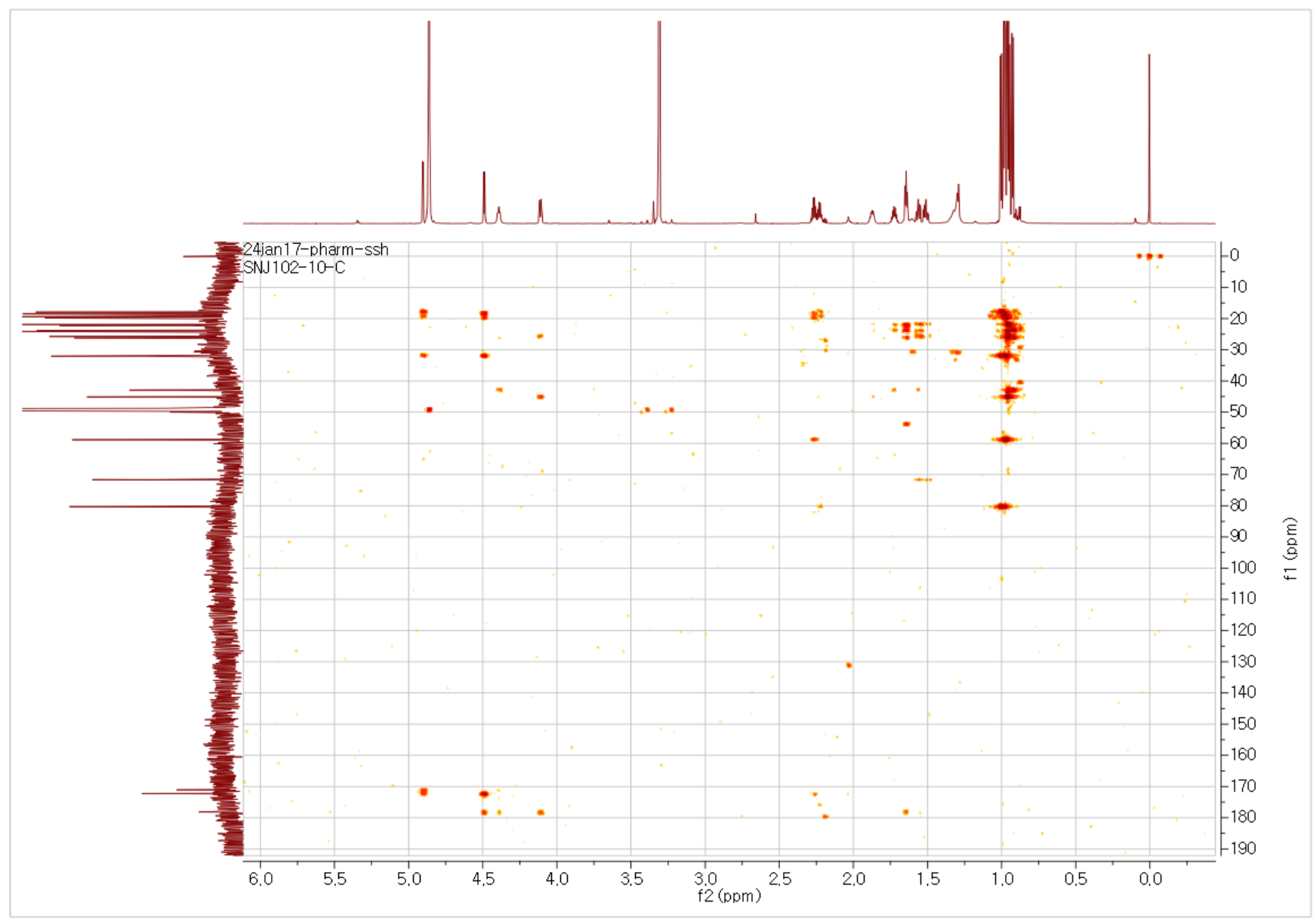


**Figure S9.** HMBC spectrum (800 MHz, CD_3_OD) of compound **1**


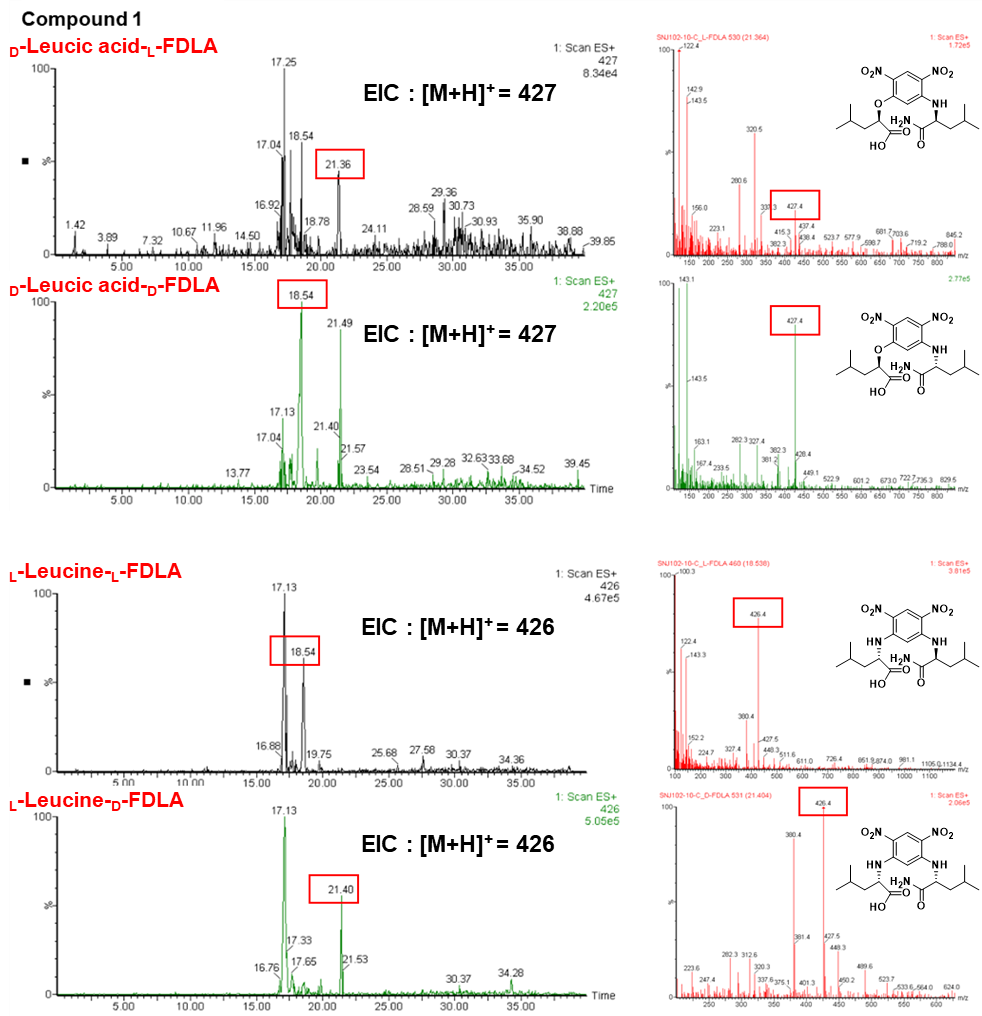


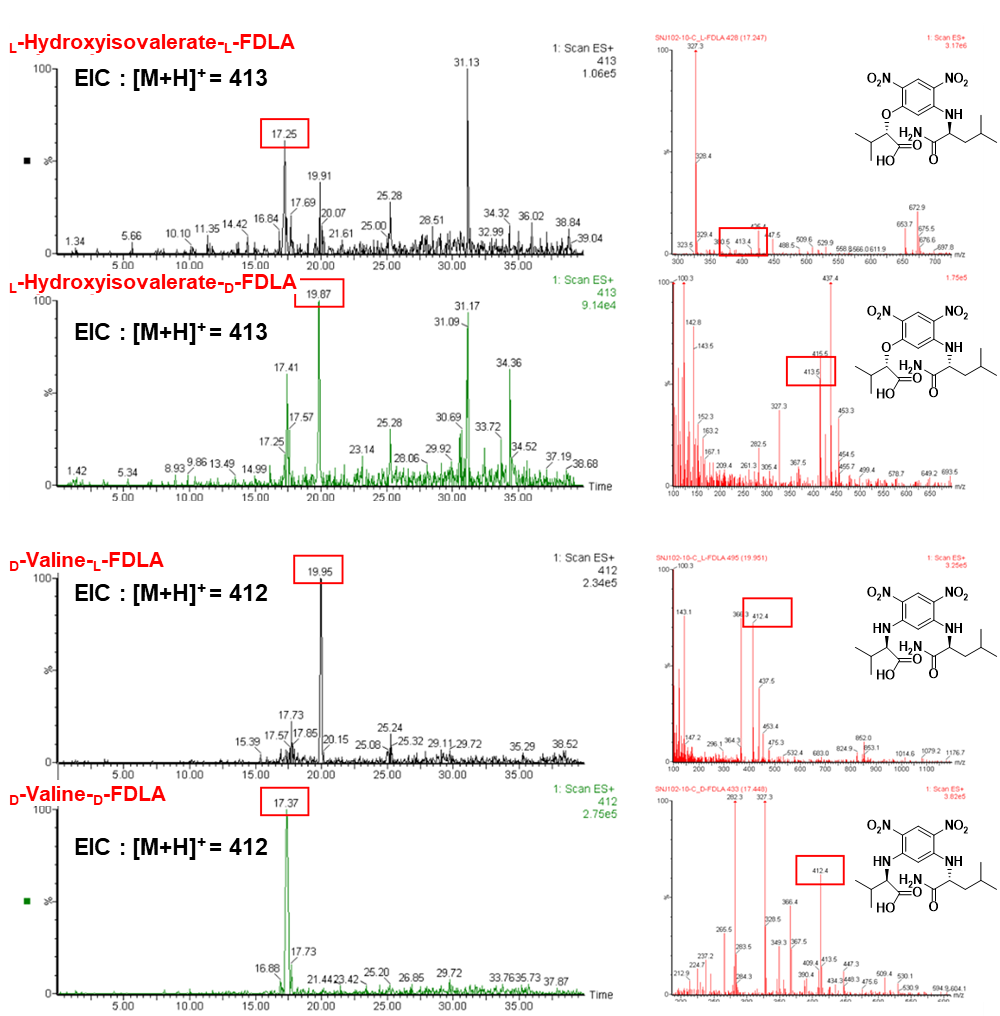


**Figure S10.** *O*-Marfey’s method analysis of compound **1**. Retention times and masses of the FDLA derivatives are indicated on LC-MS total ion chromatograms (left) and mass spectrums (right) by red boxes, respectively. The analysis indicated that compound **1** is composed of _D_-leucic acid, _D_-Valine, _L_-Hydroxyisovalerate, and _L_-Leucine.


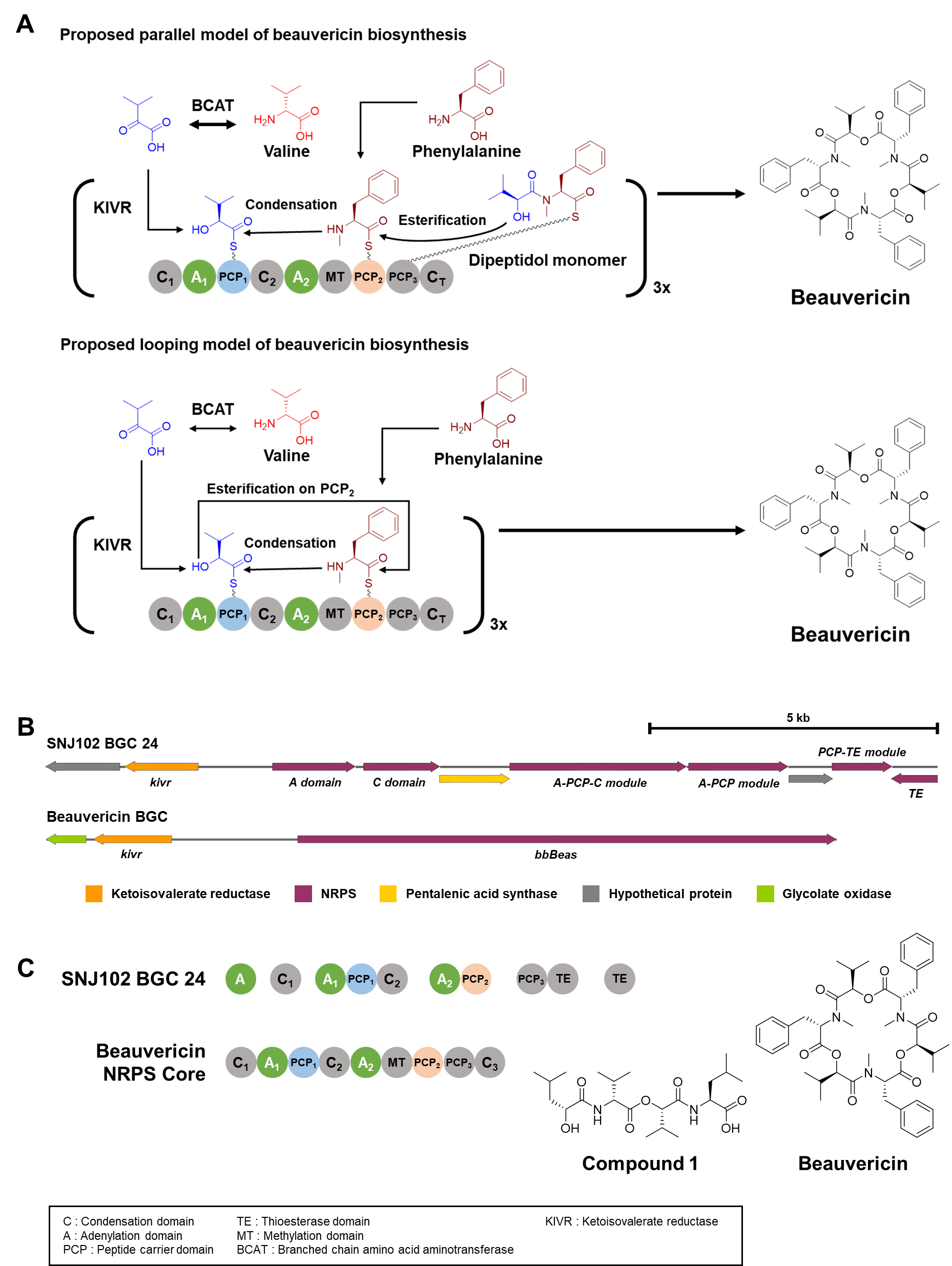


**Figure S11.** Proposed biosynthetic pathways of beauversicin and comparison of depsipeptide BGCs. **A** Propose parallel and looping model of beauvericin biosynthesis. **B** Comparison of SNJ102 depsipeptide BGC to that of beauvericin. **C** NRP modules of SNJ102 have non-canonical features, while that of beauvericin have a conventional iterative depsipeptide NRPS BGC structure.


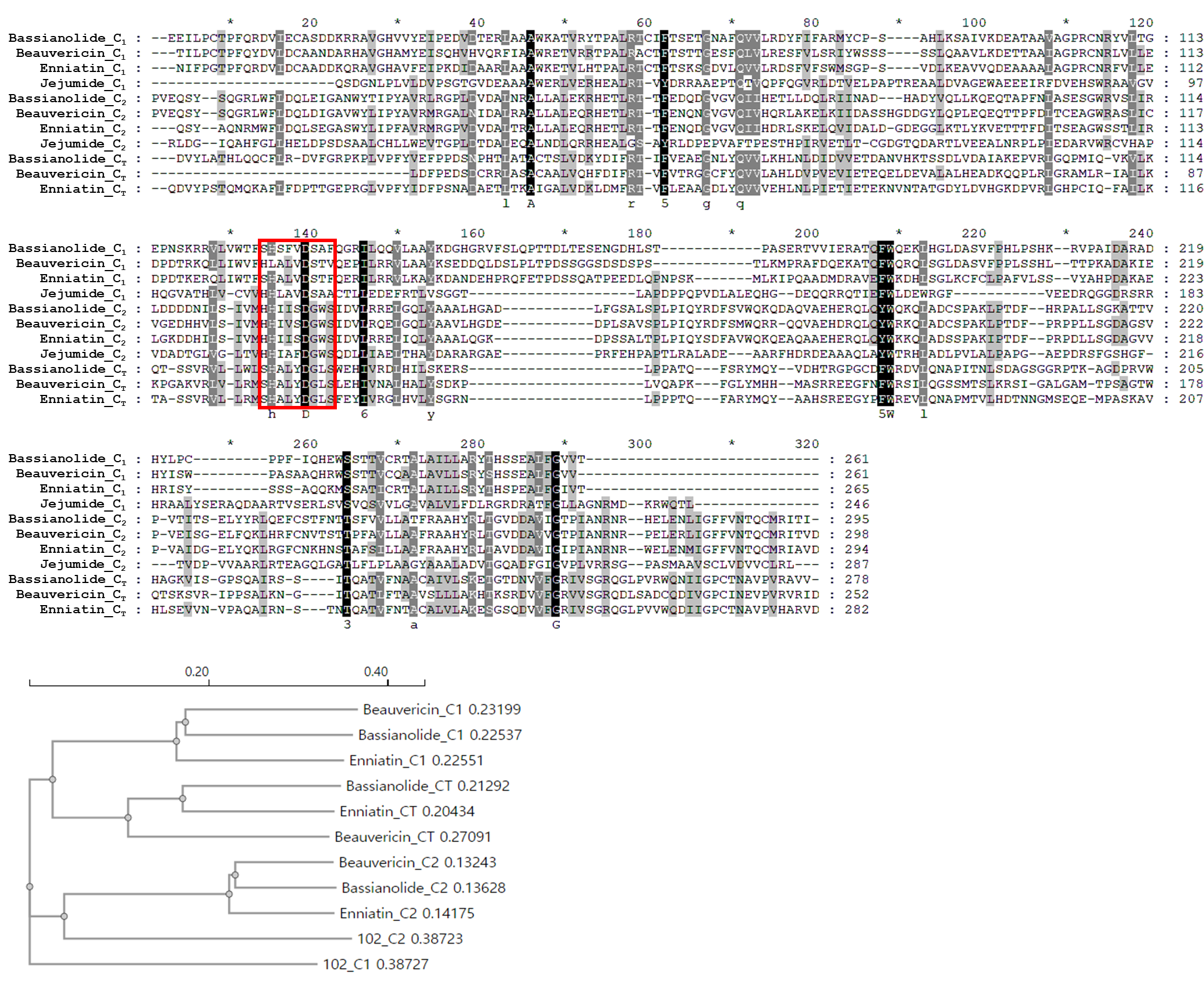


**Figure S12.** Alignments of C domains from various depsipeptide BGCs (top) and phylogenetic tree (bottom). Conserved active site residues of C domains are indicated by the red box. C_1_, C_2_, and C_T_ domains had active site motifs of HXXVDS, HHIXSDGWS, and SHALYDGLS, respectively.


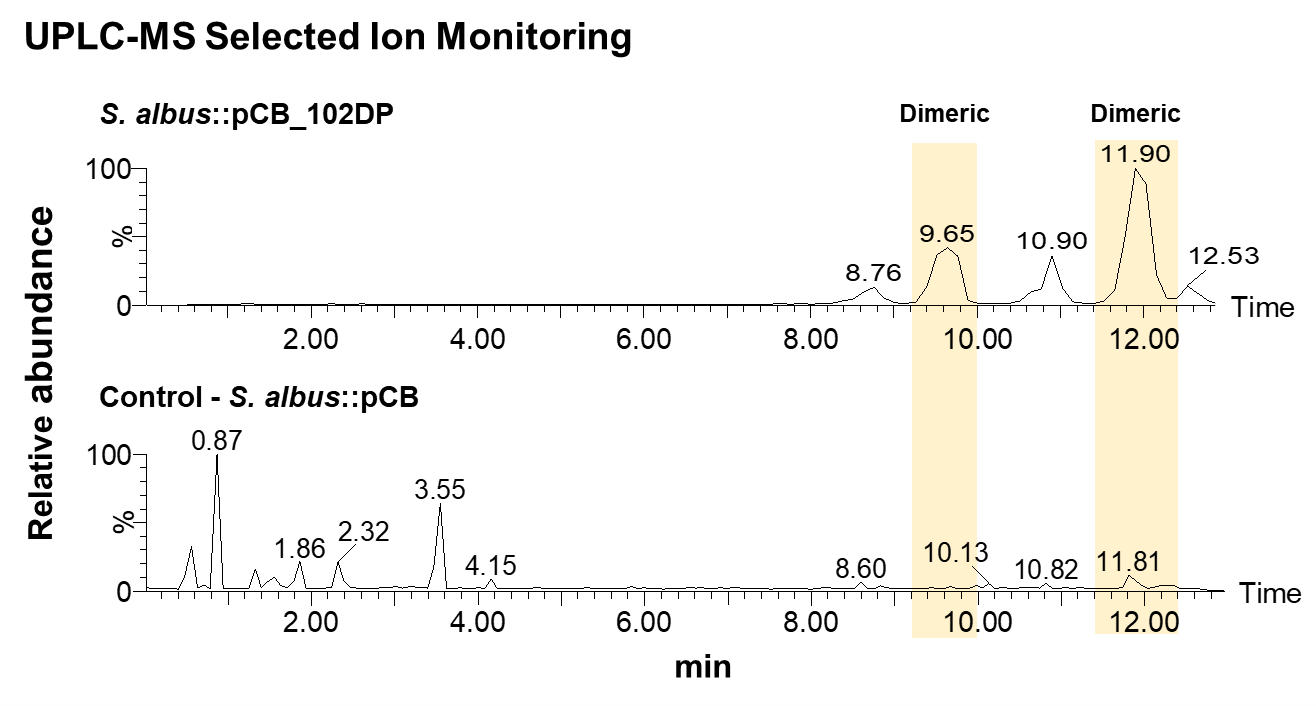


**Figure S13.** Selected ion monitoring of crude extracts analyzed by qToF LC-MS. Analysis of crude extracts from *S. albus* expressing SNJ102 BGC by qTOF LC-MS detected probable linear and cyclic dimers of compound **1** (yellow).


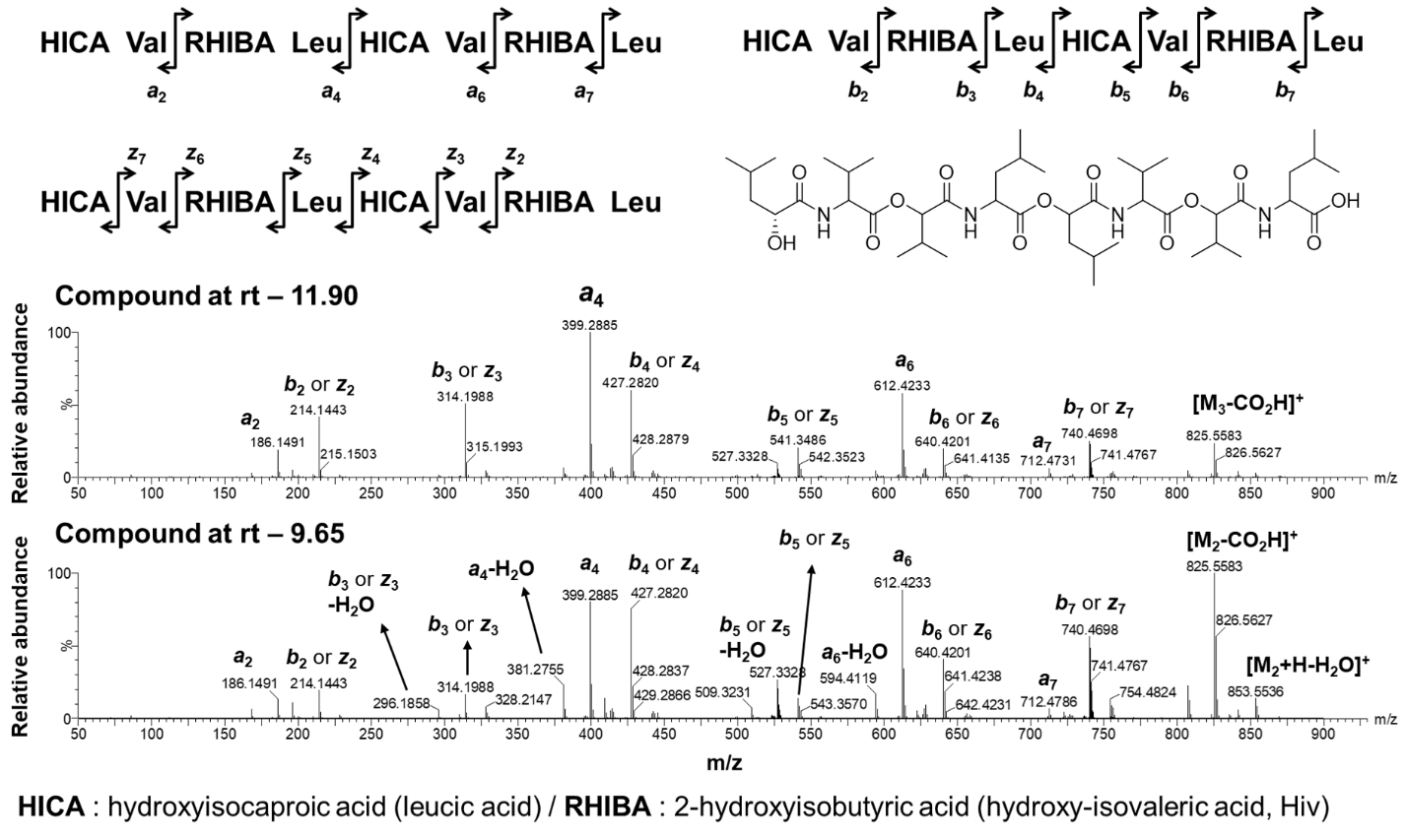


**Figure S14.** MSMS fragmentation of dimeric depsipeptide. MSMS fragmentation of dimeric depsipeptide eluted from C18 column at two different time points.

**References**

Han AR, Park SR, Park JW, Lee EY, Kim DM, Kim BG, Yoon YJ (2011) Biosynthesis of glycosylated derivatives of tylosin in *Streptomyces venezuelae*. J Microbiol Biotechnol 21: 613–616

Hassan HM, Degen D, Jang KH, Ebright RH, Fenical W (2015) Salinamide F, new depsipeptide antibiotic and inhibitor of bacterial RNA polymerase from a marine-derived *Streptomyces* sp. J Antibiot (Tokyo) 68:206–209

Igarashi Y, Shimasaki R, Miyanaga S, Oku N, Onaka H, Sakurai H, Saiki I, Kitani S, Nihira T, Wimonsiravude W, Panbangred W (2010) Rakicidin D, an inhibitor of tumor cell invasion from marine-derived *Streptomyces* sp. J Antibio (Tokyo) 63:563–565

Jang JY, Yang SY, Kim YC, Lee CW, Park MS, Kim JC, Kim IS (2013) Identification of orgamide A as an insecticidal metabolite produced by *Pseudomonas protegens* F6. J Agric Food Chem 61:6786–6791

Jung DH, Kim EJ, Jung E, Kazlauskas RJ, Choi KY, Kim BG (2016) Production of p-hydroxybenzoic acid from p-coumaric acid by *Burkholderia glumae* BGR1. Biotechnol Bioeng 113:1493–1503

Kim H, Kim JY, Ji CH, Lee D, Shim SH, Joo HS, Kang HS (2023) Acidonemycins A-C, glycosylated angucyclines with antivirulence activity produced by the acidic culture of *Streptomyces indonesiensis*. J Nat Prod 86:2039–2045

Omura S, Tanaka Y, Mamada H, Masuma R (1983) Ammonium ion suppresses the biosynthesis of tylosin aglycone by interference with valine catabolism in *Streptomyces fradiae*. J Antibiot (Tokyo) 36:1792–1794

Schubert V, Meo FD, Saaidi PL, Bartoschek S, Fiedler HP, Trouillas P, Süssmuth RD (2014) Stereochemistry and conformation of skyllamycin, a non-ribosomally synthesized peptide from *Streptomyces* sp. Acta 2897. Chemistry 20:4948–4955

Villadsen NL, Jacosen KM, Keiding UB, Weibel ET, Christiansen B, Vosegaard T, Bjerring M, Jensen F, Johannsen M, Tørring T, Poulsen TB (2016) Synthesis of *ent*-BE-43547A_1_ reveals a potent hypoxia-selective anticancer agent and uncovers the biosynthetic origin of the APD-CLD natural products. Nat Chem 9:264–272

Wirtz DA, Ludwig KC, Arts M, Marx CE, Krannich S, Barac P, Kehraus S, Josten M, Henrichgreise B, Müller A, König GM, Peoples AJ, Nitti A, Spoering AL, Ling LL, Lewis K, Crüsemann M, Schneider T (2021) Biosynthesis and mechanism of action of the cell wall targeting antibiotic hypeptin. Angew Chem Int Ed Engl 60:13579–13586

Yi JS, Yoo HW, Kim EJ, Yang YH, Kim BG (2020) Engineering *Streptomyces coelicolor* for production of monomethyl branched chain fatty acids. J Biotechnol 10:69–76
